# Characteristics, Prognosis, and Competing Risk Nomograms of Cutaneous Malignant Melanoma: Evidence for Pigmentary Disorders

**DOI:** 10.3389/fonc.2022.838840

**Published:** 2022-06-01

**Authors:** Zichao Li, Xinrui Li, Xiaowei Yi, Tian Li, Xingning Huang, Xiaoya Ren, Tianyuan Ma, Kun Li, Hanfeng Guo, Shengxiu Chen, Yao Ma, Lei Shang, Baoqiang Song, Dahai Hu

**Affiliations:** ^1^Department of Burns and Cutaneous Surgery, Xijing Hospital, Fourth Military Medical University, Xi’an, China; ^2^Department of Plastic Surgery, Xijing Hospital, Fourth Military Medical University, Xi’an, China; ^3^Department of Health Statistics, School of Public Health, Fourth Military Medical University, Xi’an, China; ^4^West China School of Public Health and West China Fourth Hospital, Sichuan University, Chengdu, China; ^5^College of Basic Medicine, Fourth Military Medical University, Xi’an, China

**Keywords:** cutaneous malignant melanoma (CMM), cause-specific death, causal inference, competing risk model, nomogram

## Abstract

**Purpose:**

Cutaneous malignant melanoma (CMM) always presents as a complex disease process with poor prognosis. The objective of the present study was to explore the influence of solitary or multiple cancers on the prognosis of patients with CMM to better understand the landscape of CMM.

**Methods:**

We reviewed the records of CMM patients between 2004 and 2015 from the Surveillance, Epidemiology, and End Results Program. The cumulative incidence function was used to represent the probabilities of death. A novel causal inference method was leveraged to explore the risk difference to death between different types of CMM, and nomograms were built based on competing risk models.

**Results:**

The analysis cohort contained 165,043 patients with CMM as the first primary malignancy. Patients with recurrent CMM and multiple primary tumors had similar overall survival status (p = 0.064), while their demographics and cause-specific death demonstrated different characteristics than those of patients with solitary CMM (*p* < 0.001), whose mean survival times are 75.4 and 77.3 months and 66.2 months, respectively. Causal inference was further applied to unveil the risk difference of solitary and multiple tumors in subgroups, which was significantly different from the total population (*p* < 0.05), and vulnerable groups with high risk of death were identified. The established competing risk nomograms had a concordance index >0.6 on predicting the probabilities of death of CMM or other cancers individually across types of CMM.

**Conclusion:**

Patients with different types of CMM had different prognostic characteristics and different risk of cause-specific death. The results of this study are of great significance in identifying the high risk of cause-specific death, enabling targeted intervention in the early period at both the population and individual levels.

## Introduction

Cutaneous malignant melanoma (CMM) is an invasive carcinoma with high mortality rates, accounting for 75% of deaths caused by skin carcinoma (1, 2). Patients with CMM are susceptible not only to recurrence (3, 4), but also to the long-term development of secondary CMM or other primary systemic cancers, such as lymphoid neoplasms and bladder carcinoma (5–7). With the great improvement in survival for CMM patients (8), patients may live long enough after the first CMM diagnosis to incur other primary cancers or noncancerous comorbidities that affect their prognosis. Given the large proportion of CMM patients with recurrent CMM or multiple primary tumors (9), the causes of death for CMM patients are complicated.

A prognostic survival model was generated for patients with invasive cutaneous melanoma in 2015 (10). However, CMM recurrence, multiple primary cancers, or other noncancerous diseases were not considered during the model development. The objective of the present study was to explore the influence of solitary CMM and CMM with multiple tumors on the survival of CMM patients to better understand the landscape of CMM. To optimally manage CMM patients by providing a quantitative prognosis of patients at the individual level, a competing risk nomogram was established (11, 12).

## Materials and Methods

### Data Sources and Processing

Patients diagnosed with skin melanoma between 2004 and 2015 were screened from the Surveillance, Epidemiology, and End Results Program (SEER). We chose SEER 18 Regs Custom Data (with additional treatment fields) for data acquisition, covering approximately 34.6% of the U.S. population (13). SEER*Stat Version 8.3.8 (NCI, Bethesda, MD) was used to extract demographics, clinical information, and outcomes. The exclusion criteria were as follows: (1) benign skin melanoma, (2) melanoma *in situ*, (3) primary diagnosis other than skin melanoma, (4) lack of positive histological results for CMM, (5) survival time of zero, (6) unknown cause of death (COD), and (7) American Joint Cancer Committee (AJCC) primary tumor grade (T) at T = 0.

The International Classification of Diseases for Oncology (3rd Edition) was used to confirm CMM. CMM patients were classified into two groups: (1) solitary CMM and (2) CMM with multiple tumors, including CMM recurrence and primary tumors after the first diagnosis of CMM. CMM recurrence was defined as newly developed CMM with the same histological type in the same region within 60 months (14), and primary tumors after the first diagnosis of CMM included newly diagnosed, non-recurrent CMM with different histological types or other primary malignant tumors. According to COD records of SEER, patient death causes were grouped into death resulting from CMM, other cancers, and noncancerous diseases (15). Missing data were imputed with the missForest method (16). To reduce the heterogeneous information of different primary tumors, only the CMM-related follow-up records were retained for model development.

### Primary Outcomes

The primary outcome was cause-specific death, including death due to CMM, death due to other cancers, and death due to noncancerous diseases.

### Data Collection

Data were collected using a pretest data collection form that included age, gender, laterality of tumor distribution, tumor subtype, tumor thickness, sentinel lymph node biopsy, ultraviolet (UV) exposure, AJCC primary tumor (T)/regional lymph node (N)/distant metastasis (M) stage, SEER stage, tumor invasion level, ulceration, regional lymph node examination, regional lymph node status, treatment, tumor size, mitotic rate, tumor grade, bone metastasis, brain metastasis, liver metastasis, lung metastasis, follow-up time, survival status, and causes of death. Variables with more than 50% missing values or with singular regression problems were discarded.

### Cumulative Incidence of Death

The cumulative incidence of death is the estimation of a person’s risk of death in a specified period. The cumulative incidence of death was calculated as the number of new deaths divided by the total number of individuals in the population at risk for a specific time interval.

### Casual Inference Method

A data-driven novel approach was performed to analyze the causal effect of solitary CMM and CMM with multiple tumors towards cause-specific death (17). Matched pairs of solitary CMM and multiple tumors were produced through an exact matching method, and the data were split into two subsamples (18). The first subsample was used for the *de novo* discovery step and the second subsample was used for the confirmation step (19). In the discovery step, the causal tree was developed to find promising subgroups denoted by *l^g^*, whereas in the confirmation step, McNemar tests were conducted to test the significance of the causal effect of subgroups in contrast with the population mean. The causal effect was measured by risk difference, the probability of death for solitary CMM minus the probability of death for CMM with multiple tumors, and the population mean, the risk difference in the total sample. Since the population mean *δ* is unknown, the confidence interval (CI) method was used to estimate the 99% CI of *δ* with *γ* = 0.01 (20). McNemar tests were applied for testing the global null hypothesis H_0_ of no effect modification in the confirmation step with *α* = 0.04 in order to achieve a total significance level of *α*+*γ* = 0.05.

### Model Development and Evaluation

Patients were randomly divided into training and validation sets at a ratio of 2:1. Backward stepwise regression based on Bayesian information criteria was conducted to select independent risk factors and avoid overfitting. A competing risk model was then constructed using the Fine and Gray proportional sub-distribution hazards regression model for different types of CMM (21, 22). Nomograms were established based on the independent risk factors to estimate the 3-, 5-, and 10-year probability of death resulting from CMM, other cancers, and noncancerous diseases. Harrell’s concordance index (*C*-index) and calibration plots were used to evaluate the model performance.

### Statistical Analysis

All statistical analysis was performed using R 4.0.5. The R packages of causalTree, mvtnorm, and rpart were used for causal inference and risk regression, while packages of crrstep and regplot were used for modeling and establishing nomograms. We used the cumulative incidence function (CIF) to illustrate the probabilities of death, and the CIF difference between categorical groups was tested using the Gray’s test (23, 24). All *p*-values resulting from the statistical testing were two-sided. *p* < 0.05 was set as statistical significance.

## Results

### Patient Characteristics

**Figure 1:**
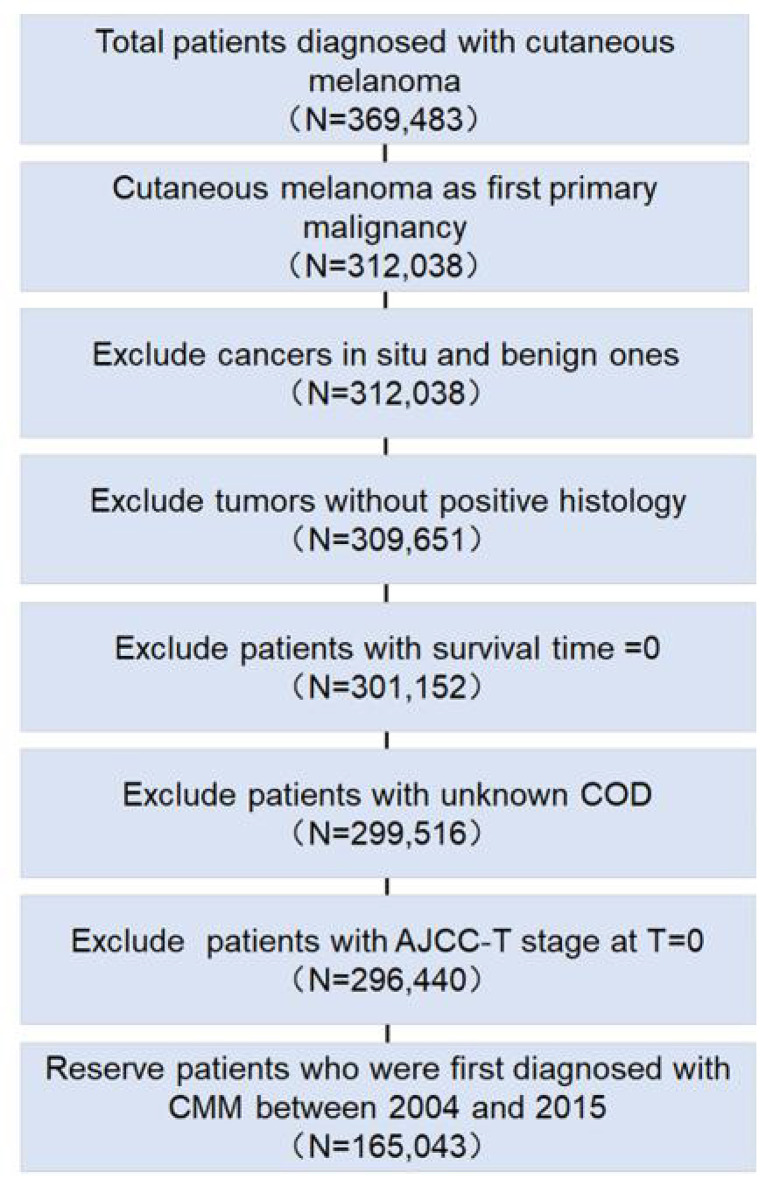
Flowchart for exclusion criteria of the clinical cohort. The number of reserved patients is demonstrated in the flowchart.

We screened total cutaneous melanoma patients with CMM in SEER databases. After excluding cases according to the eligibility criteria, the data from a total 165,043 patients, with 181,819 CMM-related records, were included in the final analysis (**Figure 1**). In **Table 1**, among the 165,043 patients diagnosed with CMM as the first primary malignancy between 2004 and 2015, 136,823 (82.9%) patients were diagnosed with solitary CMM, 2,359 (1.4%) patients with recurrent CMM, and 25,861 (15.7%) patients with multiple primary tumors. The top five detected primary tumors after first CMM diagnosis were secondary CMM (38.7%), prostate cancer (17.0%), breast cancer (8.6%), lung and bronchus cancer (6.7%), and urinary bladder cancer (4.5%). CMM patients that were male (55.1%), Caucasian (98.2%), and with high ultraviolet (UV) exposure (69.3%) were the majority (*p* < 0.001). Additionally, 44,393 patients (26.9%) underwent sentinel lymph node (SLN) biopsy, and surgery alone (93.2%) was the most common therapy for CMM patients. As of last follow-up, 11,861 patients (7.2%) died of CMM [mean survival time (MST), 32.1 months], 4,721 patients (2.9%) died of other cancers (MST, 49.1 months), and 13,850 patients (8.4%) died of noncancerous diseases (MST, 49.3 months) (**Table 1**).

**Table 1:** Demographics, clinical information, and incidence of cause-specific death of CMM patients.

Covariate	CMM patients		Total deaths		Death of CMM		Death of other cancers		Death of noncancerous diseases	
	*N*	%	*N*	%	*N*	%	*N*	%	*N*	%
**Total Patients**	165,043		30,432		11,861		4,721		13,850	–
**Diagnostic group**										
Solitary CMM	136,823	82.9	23,107	75.9	10,066	84.9	1,389	29.4	11,652	84.1
CMM recurrence	2,359	1.4	714	2.3	374	3.2	83	1.8	257	1.9
CMM-MPTs	25,861	15.7	6,611	21.7	1,421	12	3,249	68.8	1,941	14
**Gender**										
Male	92,369	56	20,438	67.2	8,089	68.2	3,232	68.5	9,117	65.8
Female	72,674	44	9,994	32.8	3,772	31.8	1,489	31.5	4,733	34.2
**Race**										
White	162,155	98.3	29,800	97.9	11,468	96.7	4,638	98.2	13,694	98.9
Black	827	0.5	287	0.9	167	1.4	42	0.9	78	0.6
Others	2,061	1.2	345	1.1	226	1.9	41	0.9	78	0.6
**UV exposure**										
High	114,329	69.3	21,488	70.6	8,306	70	3,400	72	9,782	70.6
Low	50,714	30.7	8,944	29.4	3,555	30	1,321	28	4,068	29.4
**SLN biopsy**										
Yes	44,393	26.9	8,007	26.3	4,130	34.8	1,170	24.8	2,707	19.5
No	120,650	73.1	22,425	73.7	7,731	65.2	3,551	75.2	11,143	80.5
**Treatment**										
No treatment	6,567	4	1,977	6.5	951	8	325	6.9	701	5.1
Surgery only	153,825	93.2	25,587	84.1	8,557	72.1	4,145	87.8	12,885	93
CT	2,145	1.3	1,304	4.3	1,103	9.3	108	2.3	93	0.7
RT	1,977	1.2	1,134	3.7	878	7.4	105	2.2	151	1.1
CT and RT	529	0.3	430	1.4	372	3.1	38	0.8	20	0.1
**Follow-up, months**										
Mean	68.1		42.6		32.1		49.1		49.3	
Median	62		34		25		43		43	
Range	1–155		1–155		1–151		1–150		1–155	

CMM-MPTs, multiple primary tumors after CMM first diagnosis; SLN, sentinel lymph node; CT, chemotherapy (with/without surgery); RT, radiotherapy (with/without surgery); CT and RT, chemotherapy and radiotherapy (with/without surgery).

### Cumulative Incidence of Death

Cumulative incidences of death resulting from CMM, other cancers, and noncancerous-related causes are listed in **Table 2**. Death due to CMM presented the worst 3- and 5-year probabilities of death at 5.9% and 8.1%, respectively, while the highest 10-year probabilities of death were detected in the noncancerous diseases group at 14.5%. Patients with recurrent CMM and multiple primary tumors had similar survival times with MST of 75.4 and 77.3 months, respectively (p = 0.069), both higher than that of solitary CMM (MST, 66.2 months) (*p* < 0.001).

**Table 2:** Three-, 5-, and 10-year cumulative incidences of cause-specific death among patients with CMM.

Covariate	Total deaths			p^*^	Death of CMM			p^*^	Death of other cancers			p^*^	Death of noncancerous diseases			p^*^
	3 Years (%)	5 Years (%)	10 Years (%)		3 Years (%)	5 Years (%)	10 Years (%)		3 Years (%)	5 Years (%)	10 Years (%)		3 Years (%)	5 Years (%)	10 Years (%)	
**Total Patients**	10.7	16	26.3		5.9	8.1	10.8		1.7	2.8	5.4		4.5	7.5	14.5	
**Diagnostic group**				**<0.001**				**<0.001**				**<0.001**				**<0.001**
Solitary CMM	10.8	15.6	24		6.3	8.4	10.7		0.8	1.2	1.7		4.9	7.9	14.6	
CMM recurrence	9.7	21.3	40.3		6.8	15.3	24.2		1.2	2.9	7.1		3.8	8.1	20.3	
CMM-MPTs	9.9	17.1	33.7		3.5	5.5	9.2		5.6	9.9	19.7		2.8	5.4	13.1	
**Gender**				**<0.001**				**<0.001**				**<0.001**				**<0.001**
Male	12.8	19.4	31.4		7.4	10.2	13.5		2.1	3.6	6.9		5.5	9.2	17.6	
Female	7.8	11.8	19.8		4.1	5.7	7.6		1.1	1.8	3.7		3.4	5.5	10.9	
**Race**				**<0.001**				**<0.001**				**<0.001**				**<0.001**
White	10.6	15.9	26.2		5.8	8	10.6		1.6	2.8	5.4		4.6	7.5	14.6	
Black	25.7	33.2	45		20.3	25.2	29.1		4.9	6.8	10.7		7.2	10.3	19.4	
Others	12	17	24.1		9.3	12.3	16.9		1.7	2.3	3.5		2.2	4.3	6.8	
**UV exposure**				**<0.001**				**<0.001**				**<0.001**				**<0.001**
High	10.9	16.4	27.1		6.1	8.3	11		1.8	2.9	5.7		4.7	7.7	15.1	
Low	10	15.2	24.5		5.7	7.8	10.2		1.4	2.4	4.8		4.2	7.1	13.3	
**SLN biopsy**				**0.01**				**<0.001**				**<0.001**				**<0.001**
Yes	9.7	15.7	26.7		6.6	10.1	14.4		1.4	2.4	5.3		3	5.4	11.5	
No	11	16.1	26.2		5.7	7.4	9.5		1.8	2.9	5.4		5.1	8.2	15.5	
**Treatment**				**<0.001**				**<0.001**				**<0.001**				**<0.001**
Surgery only	8.8	14.1	24.4		4.2	6.3	8.8		1.4	2.5	5.1		4.3	7.2	14.2	
CT	51.8	60.2	69.6		49.3	56.7	65.1		10.4	12.3	14.1		6.5	9.4	13.3	
RT	48.3	59.6	70.1		45.2	54	61.3		8.5	12.4	18.8		11.3	16.9	23.2	
CT and RT	75.6	82.5	85.7		74.9	80.8	83.4		22.1	28.5	33		14.5	16.1	16.1	
No treatment	24.9	30.8	40.9		16	18.5	21		5.3	6.7	9.7		9.5	13.3	21.6	

CMM-MPTs, multiple primary tumors after CMM first diagnosis; SLN, sentinel lymph node; CT, chemotherapy (with/without surgery); RT, radiotherapy (with/without surgery); CT and RT, chemotherapy and radiotherapy (with/without surgery).

^*^Difference between subgroups were tested using the Gray’s test.

The bold values represented that the p value was statistically significant.

Different cause-specific death rates were statistically associated with different types of CMM, gender, race, SLN biopsy, UV exposure, and treatment. Patients with recurrent CMM were associated with the highest probabilities of death from CMM (*p* < 0.001). The highest probabilities of death of other tumors and noncancerous causes were found in patients with multiple primary tumors (*p* < 0.001). Additionally, patients that were male, black, and with high UV exposure showed higher probabilities of all-cause death (*p* < 0.001). Patients with SLN biopsy had significantly lower probabilities of death resulting from other cancers and noncancerous diseases (*p* < 0.001), while probabilities were not decreased for patients with death from CMM. Surgery alone significantly decreased patient probabilities of any death (*p* < 0.001) (**Table 2**).

For patients with recurrent CMM, no significant difference in total death was found between single recurrence or multiple recurrences (p = 0.09). For patients with multiple primary tumors, the occurrence of different types of multiple primary tumors after first diagnosis with CMM demonstrated a different prognosis. Tumors of the lung and bronchus were associated with the worst prognosis, with 3-, 5-, and 10-year probabilities of all-cause death of 62.9%, 72.4%, and 85.2%, respectively (*p* < 0.001) (**Table S1**).

### Causal Effect of Different Types of CMM on Cause-Specific Death

Because patients with CMM recurrence and patients with multiple primary tumors have similar survival times with MST 75.4 months and 77.3 months, respectively (p = 0.069), the two types of CMM were concatenated into CMM with multiple tumors (multiple cancers of patients with CMM) for further analysis and modeling. For the estimated difference in death risk between solitary CMM and CMM with multiple tumors, the probabilities were −0.64% (95% CI, −1.6 to 0.43), −8.54% (95% CI, −9.56 to −7.46), and 2.74% (95% CI, 1.63 to 3.84) for death caused by CMM, other cancers, and noncancerous diseases, respectively.

**Figure 2:**
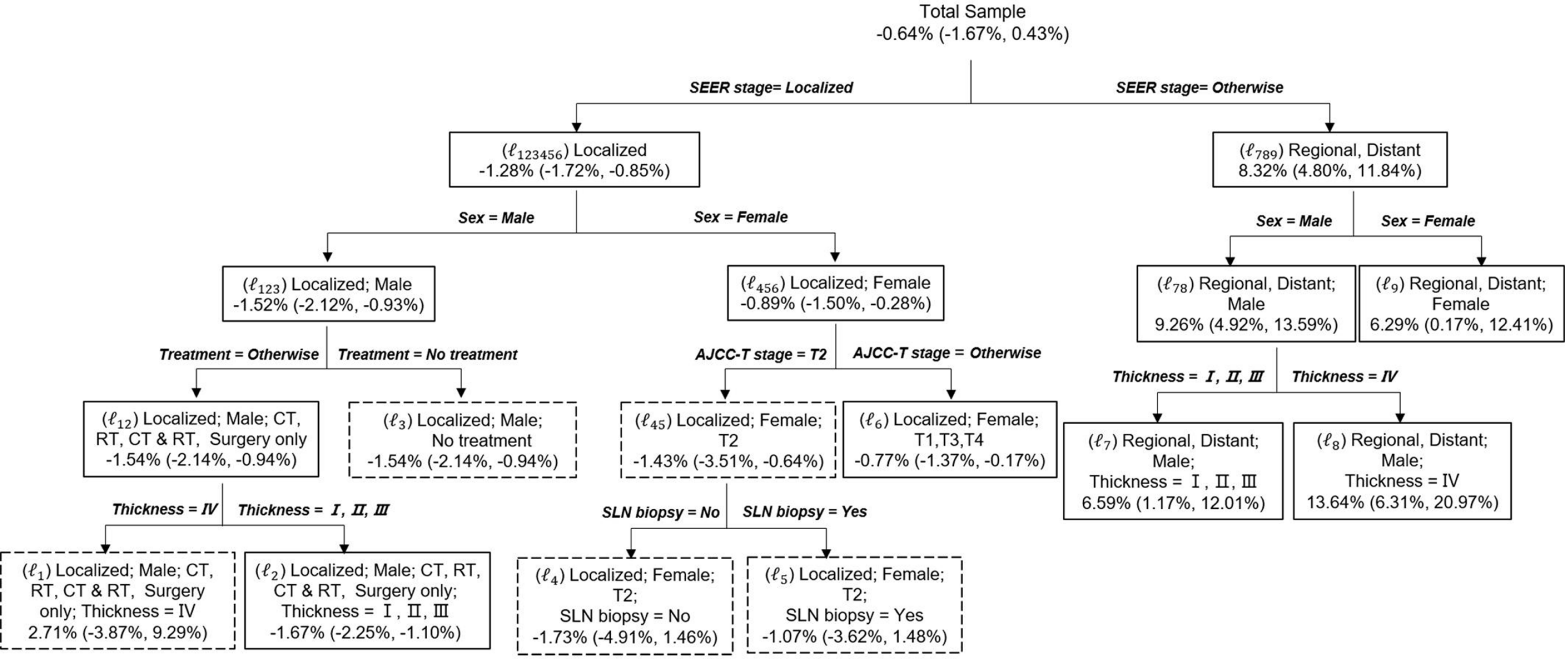
Causal Tree of patients dying of CMM. The discovered tree was developed from the first subsample and the risk difference and the 95% CI for each split subgroups calculated from the second subsample. The subgroups in solid rectangles represent a significant risk difference between solitary CMM and CMM with multiple tumors, whereas dashed rectangles indicate no significant difference. Risk difference was calculated as the probability of death for solitary CMM minus the probability of death for CMM with multiple tumors. Age: young (≤45 years), middle (45–60 years), and old (>60 years).

Prior to conducting the causal inference, the balance of matched pairs was checked, and a perfect balance was shown with an imbalance statistic (L1 = 0, LCS = 100%) (**Table S2**). For the effect modification to patients dying of CMM, the population average risk difference δwas −0.64% (99% CI, −1.67%, to 0.43). Sixteen deviates of the discovered subgroups, including terminal nodes and internal nodes, for various δ_0_ are identified in **Table 3**. The critical value was κ_a_ = 2.94 when α =0.04 and γ = 0.01. In the range of 99% CI (−1.67 to 0.43) of δ_0_, the maximum absolute deviate (D_max_) ranged from 4.15 to 5.00, all of which were larger than 2.94. Hence, there is a statistically significant effect modification in the entire population, which made the risk difference of cause-specific deaths of sensitive or vulnerable populations significantly different than that of the overall population (*p* < 0.05). The subgroup analysis showed that for solitary CMM patients, those with localized CMM, male, accepted treatments, and tumor thickness ≤ 400 mm had a significantly lower risk of death −1.67% (99% CI, −2.25 to −1.10%) (*p* < 0.05). In contrast, for CMM patients with multiple cancers, male and tumor thickness level > 400 mm had a significantly higher risk of death 13.64% (99% CI, 6.31 to 20.97) (*p* < 0.05) (**Figure 2**). Similarly, the risk difference of solitary CMM and CMM with multiple tumors dying from other cancers and noncancerous diseases, illustrated in **Tables S3**, **S4** and **Figures S1**, **S2**, all showed significant effect modification in the entire population and heterogeneous effects between the two types of CMM among vulnerable subgroups.

**Table 3:** McNemar tests of the null hypothesis of no effect modification for solitary CMM and CMM with multiple tumors on patients dying of CMM.

	Subgroups																
	ℓ1	ℓ2	ℓ3	ℓ4	ℓ5	ℓ6	ℓ7	ℓ8	ℓ9	ℓ12	ℓ45	ℓ78	ℓ123	ℓ456	ℓ789	ℓ123456	
*δ*	D1	D2	D3	D4	D5	D6	D7	D8	D9	D10	D11	D12	D13	D14	D15	D16	Dmax
−0.0167	1.12	0	0.16	−0.03	0.26	1.28	2.69	3.79	2.29	0.24	0.14	4.48	0.27	1.2	5	0.94	5
−0.0115	0.97	−0.97	0	−0.28	0.04	0.53	2.52	3.65	2.15	−0.73	−0.19	4.26	−0.72	0.39	4.74	−0.34	4.74
−0.0062	0.89	−1.94	−0.16	−0.47	−0.19	−0.21	2.35	3.5	2.02	−1.7	−0.48	4.03	−1.69	−0.41	4.48	−1.6	4.48
−0.001	0.73	−2.89	−0.4	−0.72	−0.41	−0.95	2.18	3.36	1.82	−2.66	−0.82	3.81	−2.68	−1.22	4.19	−2.88	4.19
0.0043	0.58	−3.86	−0.56	−0.91	−0.64	−1.7	2.01	3.29	1.68	−3.64	−1.11	3.64	−3.67	−2.02	3.97	−4.15	4.15
	**Subgroups**															**Total**	
Proportion (%)	1.7	53.9	2.2	3.5	2.8	28.9	2.9	1.8	2.2	55.6	6.4	4.7	57.8	35.3	6.9	93.1	100
Solitary CMM (%)	23.2	3.8	8.4	8.3	3.7	2.2	32.8	43.5	29.6	4.4	6.3	36.8	4.6	2.9	34.5	3.9	6.0
Multiple tumors (%)	20.5	5.5	9.5	10.1	4.8	2.9	26.2	29.8	23.3	6.0	7.7	27.6	6.1	3.8	26.2	5.2	6.7
Risk Difference (%)	0.0	0.0	0.0	0.0	0.0	0.0	0.1	0.1	0.1	0.0	0	0.1	0.0	0	0.1	0.0	-0.7
Odds ratio	1.2	0.7	0.9	0.8	0.8	0.7	1.4	1.8	1.4	0.7	0.8	1.5	0.7	0.8	1.5	0.7	1.0

Sixteen deviates from the subgroups with the maximum absolute deviate where the critical values D_T_ = 2.94 when α = 0.04 and γ = 0.01.

### Competing Risk Nomogram

**Figure 3:**
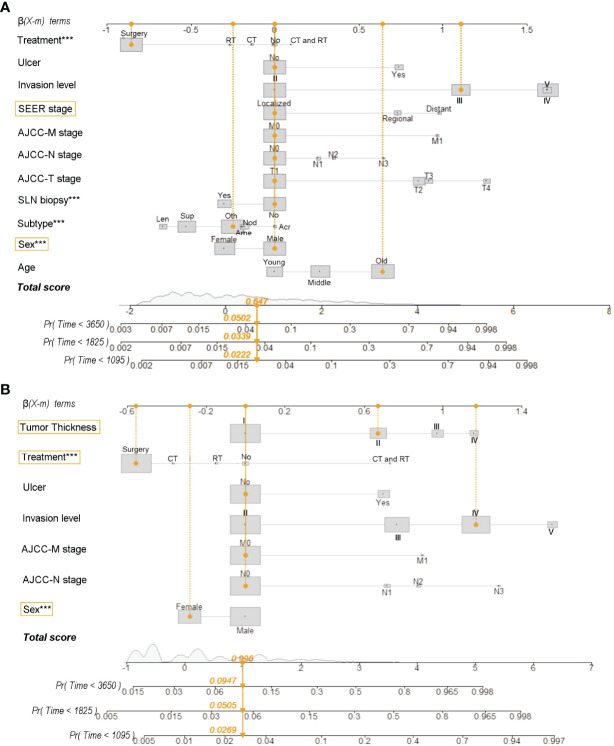
Nomograms for predicting 3-, 5-, and 10-year probabilities of death resulting from CMM for patients with solitary CMM **(A)** and CMM with multiple tumors **(B)**. Treatment: No, No treatment; CT, chemotherapy (with/without surgery); RT, radiotherapy (with/without surgery); CT and RT, chemotherapy and radiotherapy (with/without surgery). Subtype (histological): Acr, Acral lentiginous CMM; Ame, Amelanotic CMM; Len, Lentigo CMM; Nod, Nodular CMM; Sup, Superficial spreading CMM; Oth, Other uncommon CMM. Tumor thickness: I, ≤100 mm; II, 100–200 mm; III, 200–400 mm; IV, >400 mm. Age: young (≤45 years), middle (45–60 years), and old (>60 years). Reg, regional; LN, lymph node; SLN, sentinel lymph node. ***Statistical significance was less 0.001.

**Figure 4:**
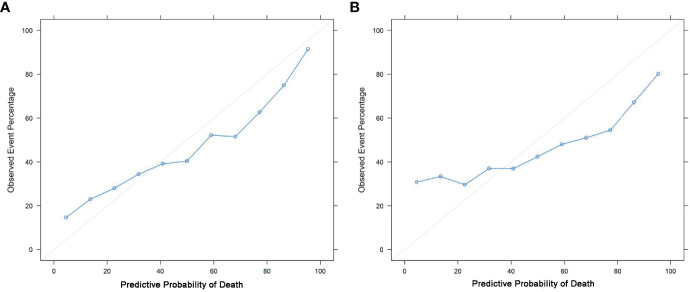
Calibration curves. Calibrations of the probabilities of CMM-caused death among patients with solitary CMM **(A)** and CMM with multiple tumors **(B)** based on validation sets.

The nomograms were constructed based on the competing risk model with the training data set, to predict 3-, 5-, or 10-year probabilities of cause-specific death (**Figure 3**). In the external validation cohort, the discrimination (C-index) to death due to CMM, other cancers, and noncancerous diseases were 0.86 (95% CI, 0.84 to 0.88), 0.82 (95% CI, 0.81 to 0.83), and 0.58 (95% CI, 0.57 to 0.61) for solitary CMM, and 0.79 (95% CI, 0.76 to 0.82), 0.61 (95% CI, 0.56 to 0.66), 0.58 (95% CI, 0.53 to 0.63) for CMM with multiple tumors, respectively. We observed that age, gender, ulcer, AJCC-T, N, M stages, SLN biopsy, histological types, and invasion level were significantly associated with the probabilities of death from CMM with a solitary tumor (**Table S5**; **Figure 3A**). In contrast, for CMM with multiple tumors, age, ulcer, tumor thickness, AJCC-N, M stages, and invasion level recorded at the patient’s first diagnosis were independent prognostic risk factors of CMM-caused death (**Table S6**; **Figure 3B**). The calibration curves represented an ideal agreement in the observed and predicted outcomes of patients dying of CMM (**Figure 4**). Additionally, the prognostic factors and predicted probabilities of death of other cancers and noncancerous diseases across different types of CMM are shown in **Tables S7****–****S10** and **Figures S3**, **S4**.

## Discussion

To the best of our knowledge, the present study is the first systematic population-based investigation of the long-term cause-specific death of different types of CMM. We found that different cause-specific death rates were statistically associated with different types of CMM, gender, race, SLN biopsy, UV exposure, and treatments. According to our causal reference analysis, solitary CMM and CMM with multiple tumors had causal relationships with differences in patient outcomes. The nomograms to predict 3-, 5-, or 10-year probabilities of death resulting from CMM were constructed based on a competing risk model and validated by validation set.

Cutaneous melanoma causes 55,500 deaths annually. The incidence and mortality rates of the disease differ widely across the globe depending on access to early detection and primary care (25–27). Patients with recurrent CMM and multiple primary tumors had similar overall survival time with MST of 75.4 and 77.3 months, which was significantly higher than that of solitary CMM (*p* < 0.001). This difference implied that most patients with a relatively long survival time suffered from CMM with multiple tumors; therefore, solitary CMM and CMM with multiple tumors should be treated separately when evaluating CMM patient prognosis. According to our findings, death of CMM and noncancerous causes are the common outcome of the patient with solitary tumor and CMM recurrence, while patients with multiple primary tumors mainly died of other cancers. On the basis of patient cause-specific death of different types of CMM, the development of other primary tumors (such as lung and bronchus tumors) and systemic noncancerous diseases detected after 5 years from first diagnosis of CMM should take greater attention and more positive curation.

In terms of the diagnosis of CMM patients, we found that SLN biopsy was related to good prognosis in all-cause death, other cancer death, and other noncancerous-disease death (*p* < 0.05), but not in death due to CMM. A possible rationale is that the procedure of SLN biopsy can provide valuable information for following treatment, and may reduce distant metastasis and CMM recurrence to some degree (28, 29). For treatment strategies in CMM, we found that surgery alone was a beneficial factor for the prognosis of CMM patients. This finding was consistent with surgery greatly decreasing patient probability of death (30). Comparing the probability of death with the control groups, patients dying of different causes who received SLN biopsy or treatment by surgery alone demonstrated different or even opposite influence on patient outcomes (*p* < 0.001). Therefore, a detailed exploration of the complex outcomes and prognosis associated with various types of CMM is important to improve the understanding of CMM.

Consistent with some previous studies (5, 31), the present population-based analysis found that solitary CMM and CMM with multiple tumors were associated with differences in patient outcomes. We conducted causal inference to confirm this relationship without bias or other confounding factors. Under the control of 16 covariates, patients were partitioned by causal tree into vulnerable subgroups in the first subsample and were tested in the second subsample, unlike normal practices that analyze the causation using the same sample. In addition, the CI method was used to estimate the population mean with a 99% CI. Therefore, the sample average was not assumed to be exactly equal to the population mean. Our approach considered the alternative hypothesis to be significant when all deviates corresponding to values of population average lying in the range of the 99% CI were greater than the critical value, instead of using *p*-value to test the hypothesis. These methodological improvements should help us obtain more solid evidence on the causalities between different types of CMM and patient cause-specific death with powerful statistical tests. Our work implies that solitary CMM and CMM with multiple tumors significantly influence patient outcomes and the risk difference of cause-specific death in subgroups. Thus, solitary tumor and multiple tumors should be studied separately on prognostic research of CMM in the future. In practice, the treatment and surveillance of those patients should be different. Although the causal inference method could explicitly unveil the effect modification of different types of CMM on patient outcomes and identify vulnerable groups at the population level (32), comprehensive nomograms could provide estimation of the cumulative incidence of death at an individual patient level (33).

Given the relatively indolent progression of CMM with improved treatment strategies, patients are more likely to suffer from other diseases (34–36). Therefore, competing causes of death have become a critical consideration when evaluating probabilities of death. To the best of our knowledge, the present study supplies the first comprehensive competing risk nomogram for both solitary CMM and CMM with multiple tumors based on a large cohort without being subject to selection and referral biases (37, 38). Across patients with solitary CMM and CMM with multiple tumors, nomograms provide accurate absolute risk estimates individually for patient death of CMM and other cancer, with C-index >0.6. Interestingly, although thousands of patients were diagnosed with solitary tumors, they unexpectedly died of other cancers, and our model has a great discriminative ability with a c-index of 0.82 (99% CI, 0.81 to 0.83). These results imply that the predictive model could assist in discovering undetectable mortal tumors clinically to further reduce the occurrence of missed diagnoses. Quantitative estimation of an individual’s prognosis could help clinicians assess disease progression, select treatment, and stratify participants in clinical trials (39)

Significant differences in prognostic factors between solitary CMM and CMM with multiple tumors associated with patient risk of death were detected in our work, and some risk factors overlapped in the partitioned subgroups and competing risk model. For instance, in patients dying of CMM, gender and SEER stages were shared factors in solitary CMM, and gender, treatment, and tumor thickness were shared factors in CMM with multiple tumors. These overlaps could be the most important and conservative factors in the discrimination of high-risk groups resulting from different causes and in the evaluation of patient prognosis. Previously, the most demonstrated prognostic factors in nomograms were also reported to greatly affect the prognosis of some specific CMM populations (40, 41). The AJCC staging system has been the most common tool to evaluate patient prognosis in clinical practice (42), while our population-based nomograms incorporate patient demographics and other clinical information with AJCC stages, to contribute to more credible prognostic models. Some limitations of the study include missing data due to the exclusion criteria, the necessity for further external validation, and the limited ability of nomograms in prediction probabilities of death due to noncancerous causes.

## Conclusion

Patients with solitary CMM and CMM with multiple tumors had different prognostic characteristics and different risk of cause-specific death. Solitary CMM and CMM with multiple tumors had causal relationships with differences in patient outcomes, and nomograms to predict 3-, 5-, or 10-year probabilities of death resulting from cause-specific death were constructed. According to causalTree and nomograms, we identified that sex and SEER stage for the solitary CMM group, and sex, tumor thickness, and treatment for the CMM with multiple tumors group are the mostly important factors for patients’ outcome. Hence, the results are significant for identifying the high risk of cause-specific death and for targeted intervention in the early period at both the vulnerable population and individual levels.

## Data Availability Statement

The original contributions presented in the study are included in the article/**Supplementary Material**. Further inquiries can be directed to the corresponding authors.

## Author Contributions

ZL, XL, DH, BS and LS contributed to the study and experimental design. XY, TL, ZL, and XH contributed to data downloading. XL, XR, and TM contributed to data preprocessing. ZL, XL, and XY contributed to missing data imputation. ZL, XL, XY, KL, and HG contributed to model development and performance evaluation. ZL, SC, and YM contributed to additional data analysis. DH, BS and LS contributed to proofreading and critical revision. ZL, XL, and XY contributed to writing the manuscript. All authors contributed to the article and approved the submitted version.

## Funding

This study was funded by the National Natural Science Foundation of China (No. 81772071). The funding body had no influence on the study design, data preparation, statistical analysis, and manuscript writing.

## Conflict of Interest

The authors declare that the research was conducted in the absence of any commercial or financial relationships that could be construed as a potential conflict of interest.

## Publisher’s Note

All claims expressed in this article are solely those of the authors and do not necessarily represent those of their affiliated organizations, or those of the publisher, the editors and the reviewers. Any product that may be evaluated in this article, or claim that may be made by its manufacturer, is not guaranteed or endorsed by the publisher.
