# Supplementary material for: Characteristics, Prognosis, and Competing Risk Nomograms of Cutaneous Malignant Melanoma: Evidence for Pigmentary Disorders
Source: Front Oncol. 2022 Jun 1;12:838840. doi: 10.3389/fonc.2022.838840 (PMC9198425; doi:10.3389/fonc.2022.838840)

**Figure S1**. Causal Tree of patients dying of other cancers. The discovered tree was developed from the first subsample and the risk difference and the 95% CI for each split subgroups calculated from the second subsample. The subgroups in solid rectangles represent a significant risk difference between solitary CMM and CMM with multiple tumors; whereas, dashed rectangles indicate no significant difference. Risk difference was calculated as the probability of death for solitary CMM minus the probability of death for CMM with multiple tumors. Age: young (≤45 years), middle (45-60 years), old (>60 years).


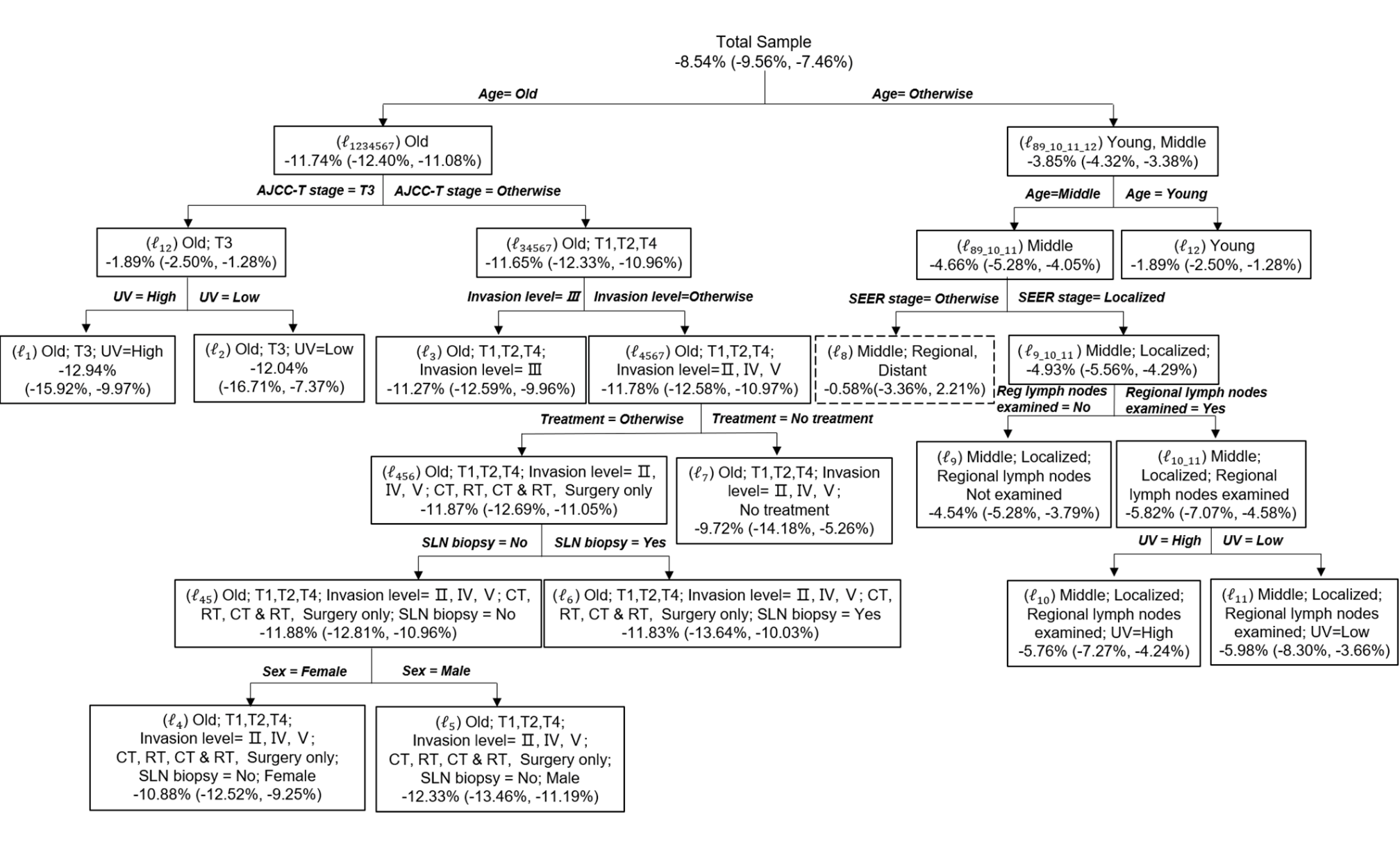


**Figure S2**. Causal Tree of patients dying of noncancerous diseases. Age: young (≤45 years), middle (45-60 years), old (>60 years).


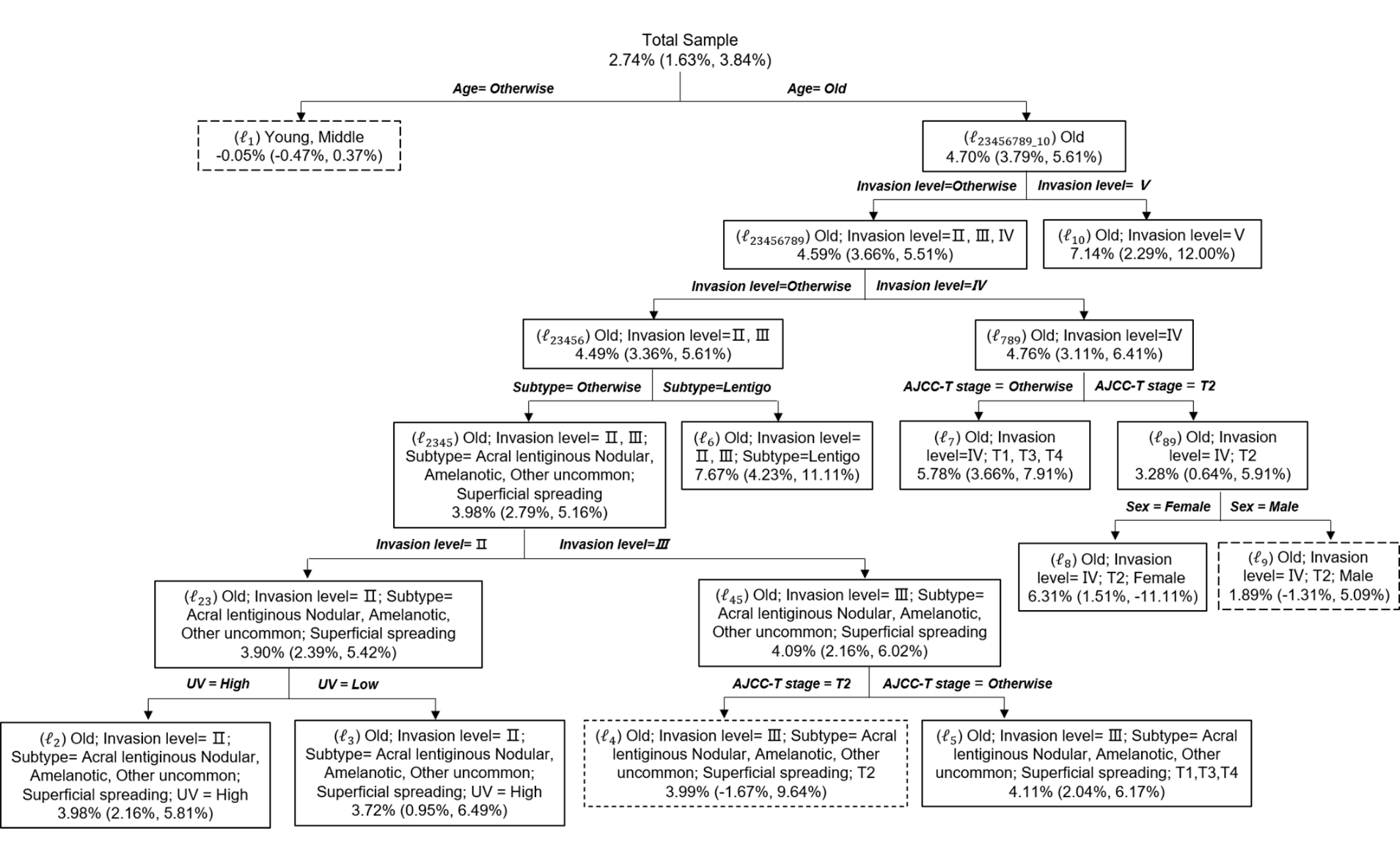


**Figure S3**. Nomograms for predicting 3-, 5-, and 10-year probabilities of death resulting from other cancers for patients with solitary CMM (A) and CMM with multiple tumors (B). Treatment: No, No treatment; CT, chemotherapy (with/without surgery); RT, radiotherapy (with/without surgery); CT and RT, chemotherapy and radiotherapy (with/without surgery). Subtype (histological): Acr, Acral lentiginous CMM; Ame, Amelanotic CMM; Len, Lentigo CMM; Nod, Nodular CMM; Sup, Superficial spreading CMM; Oth, Other uncommon CMM. Tumor thickness: I, ≤100mm; II, 100-200mm; III, 200-400mm; IV, >400mm. Age: young (≤45 years); middle (45-60 years); old (>60 years).

Abbreviations: Reg, regional; LN, lymph node; SLN, sentinel lymph node


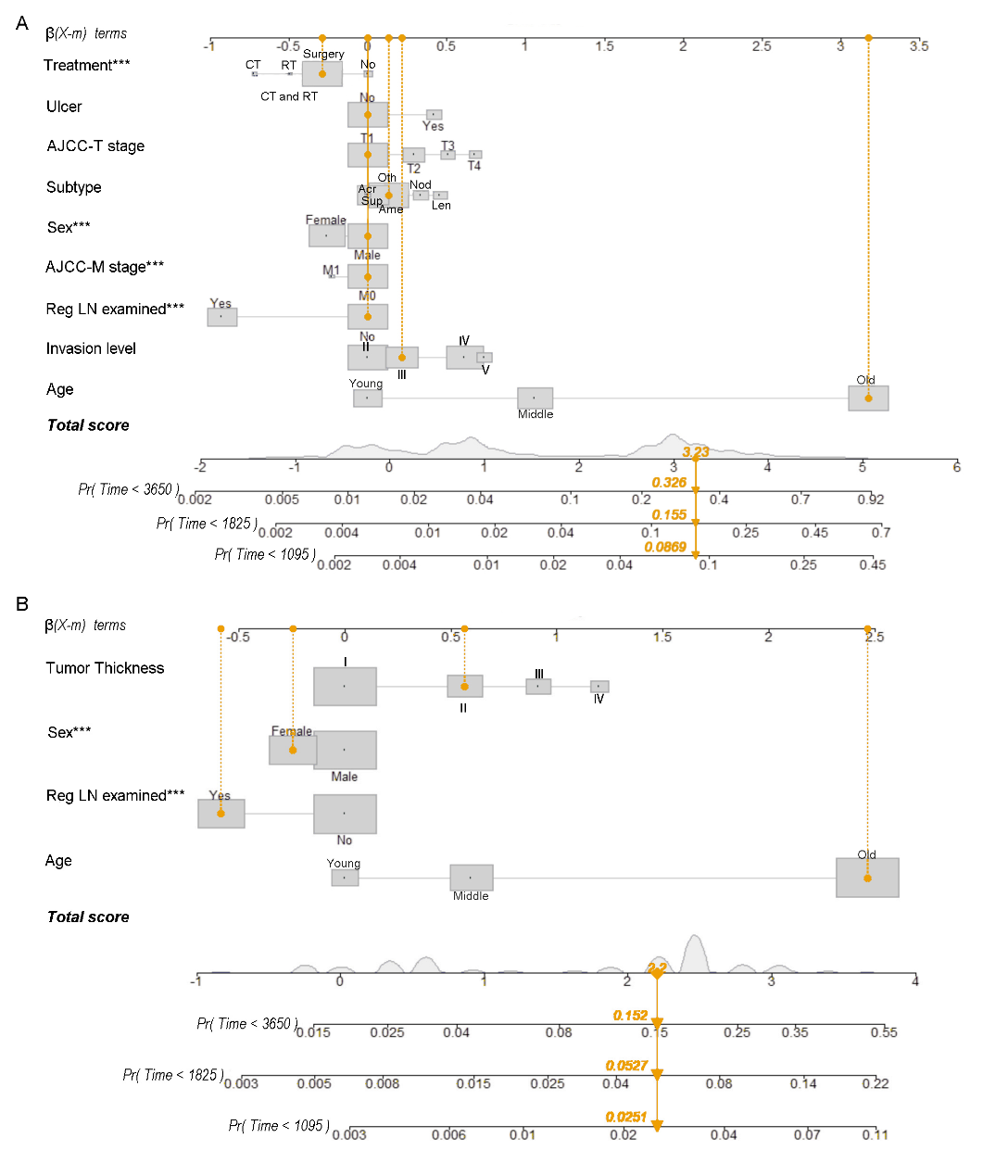


**Figure S4**. Nomograms for predicting 3-, 5-, and 10-year probabilities of death resulting from noncancerous diseases. for patients with solitary CMM (A) and CMM with multiple tumors (B).


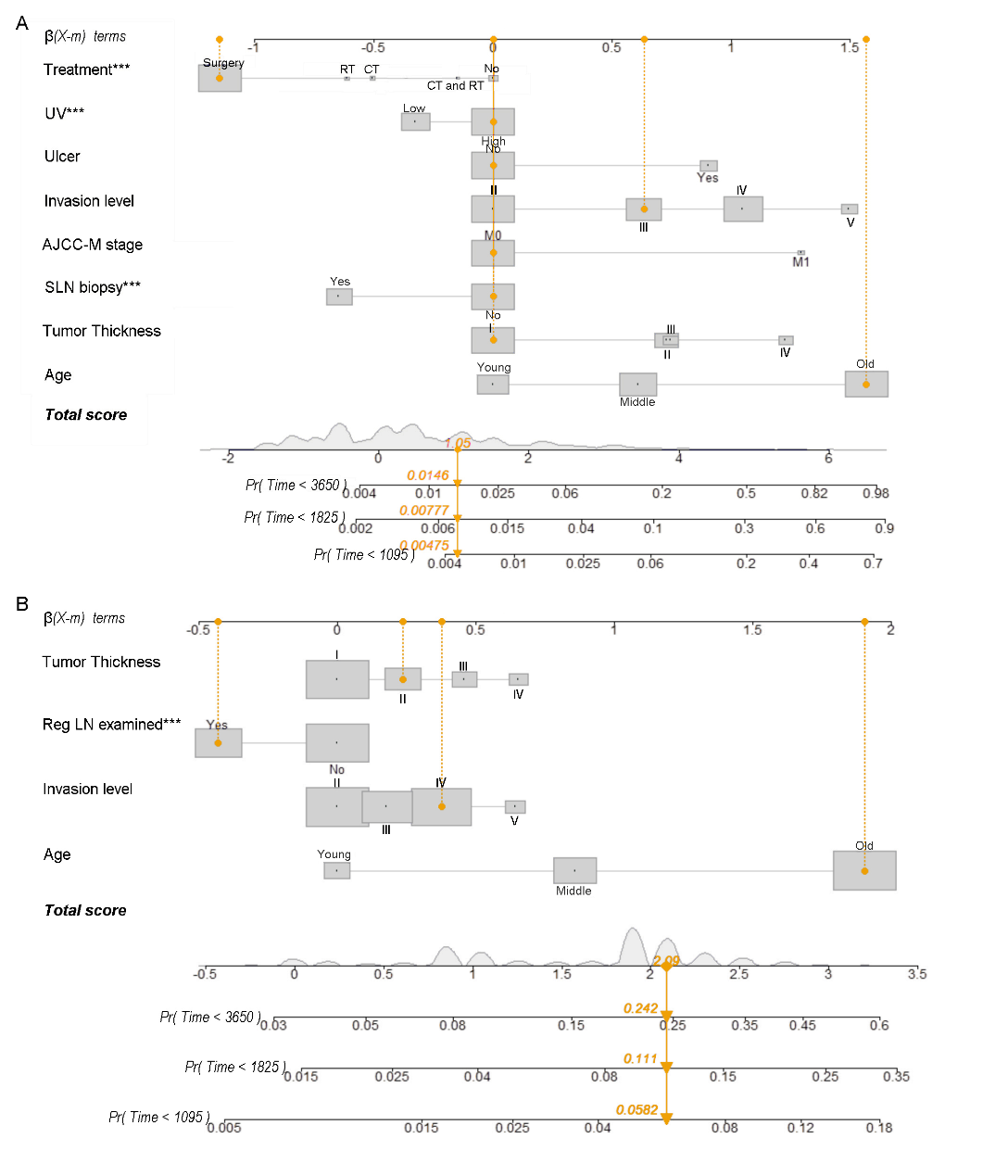

Supplement: Supplementary file 1 [file DataSheet_1.docx]
