# Supplementary material for: Characteristics, Prognosis, and Competing Risk Nomograms of Cutaneous Malignant Melanoma: Evidence for Pigmentary Disorders
Source: Front Oncol. 2022 Jun 1;12:838840. doi: 10.3389/fonc.2022.838840 (PMC9198425; doi:10.3389/fonc.2022.838840)
Supplement: Supplementary file 2 [file Table_1.docx]

| **Diagnosis group** | | **Total deaths** | | |  | **Death of CMM** | | |  | **Death of other cancers** | | |  | **Death of noncancer-diseases** | | |  |
| --- | --- | --- | --- | --- | --- | --- | --- | --- | --- | --- | --- | --- | --- | --- | --- | --- | --- |
|  |  | 3 Years  (%) | 5 Years  (%) | 10 Years  (%) | *P^*^* | 3 Years  (%) | 5 Years  (%) | 10 Years  (%) | *P^*^* | 3 Years  (%) | 5 Years  (%) | 10 Years  (%) | *P^*^* | 3 Years  (%) | 5 Years  (%) | 10 Years  (%) | *P^*^* |
| **Total Patients** | | 10.7 | 16 | 26.3 |  | 5.9 | 8.1 | 10.8 |  | 1.7 | 2.8 | 5.4 |  | 4.5 | 7.5 | 14.5 |  |
| **Solitary CMM** | | 10.8 | 15.6 | 24 |  | 6.3 | 8.4 | 10.7 |  | 0.8 | 1.2 | 1.7 |  | 4.9 | 7.9 | 14.6 |  |
| **CMM recurrence** | |  |  |  | 0.09 |  |  |  | 0.93 |  |  |  | 0.59 |  |  |  | **0.04** |
|  | once | 10.6 | 22.5 | 40.7 |  | 7.3 | 15.8 | 23.4 |  | 1.2 | 2.7 | 7.5 |  | 4.4 | 9.3 | 21.2 |  |
|  | More than once | 6.3 | 16.9 | 38.2 |  | 4.9 | 13.4 | 25.6 |  | 1.2 | 3.5 | 6.2 |  | 1.6 | 3.7 | 16.8 |  |
| **CMM-MPTs** | |  |  |  | **<0.001** |  |  |  | **<0.001** |  |  |  | **<0.001** |  |  |  | **<0.001** |
|  | Melanoma of the Skin | 18.1 | 25.4 | 37.3 |  | 11.3 | 14.1 | 17.7 |  | 3.3 | 5.5 | 8.7 |  | 6.8 | 10.9 | 19.7 |  |
|  | Prostate | 11.1 | 18.8 | 34.9 |  | 2.7 | 4 | 7.7 |  | 5.7 | 9.8 | 17.1 |  | 4.4 | 8.3 | 17.7 |  |
|  | Breast | 12.1 | 19.8 | 37.3 |  | 3.8 | 4 | 5.9 |  | 7 | 12.4 | 21.5 |  | 3 | 6.5 | 17.8 |  |
|  | Lung and Bronchus | 62.9 | 72.4 | 85.2 |  | 13.8 | 17.1 | 22.9 |  | 60.3 | 69.7 | 77.3 |  | 16.2 | 19.8 | 42.9 |  |
|  | Urinary Bladder | 31.2 | 41.9 | 58.2 |  | 5.8 | 7.3 | 12.3 |  | 20.7 | 27.8 | 30.4 |  | 14.9 | 21.3 | 38.8 |  |

**Table S1**. 3-, 5-, and 10-year cumulative incidences of cause-specific death among patients with CMM. Different frequencies of CMM recurrence and top five multiple primary tumors after CMM first diagnosis were studied separately.

Abbreviations: CMM-MPTs, multiple primary tumors after CMM first diagnosis.
