# Supplementary material for: Characteristics, Prognosis, and Competing Risk Nomograms of Cutaneous Malignant Melanoma: Evidence for Pigmentary Disorders
Source: Front Oncol. 2022 Jun 1;12:838840. doi: 10.3389/fonc.2022.838840 (PMC9198425; doi:10.3389/fonc.2022.838840)
Supplement: Supplementary file 3 [file Table_2.docx]

|  | statistic | type | L1 |
| --- | --- | --- | --- |
| Age | 4.19E+03 | (Chi2) | 9.02E-17 |
| Tumor Thickness | 0.00E+00 | (diff) | 1.39E-16 |
| Gender | 1.28E+03 | (Chi2) | 8.33E-17 |
| Laterality | 1.67E+00 | (Chi2) | 1.73E-16 |
| Subtype | 3.79E+02 | (Chi2) | 3.86E-17 |
| SLN biopsy | 1.92E+01 | (Chi2) | 1.39E-16 |
| UV exposure | 3.62E-03 | (Chi2) | 5.55E-17 |
| AJCC-T stage | 1.76E+02 | (Chi2) | 1.35E-16 |
| AJCC-N stage | 1.41E+02 | (Chi2) | 2.25E-16 |
| AJCC-M stage | 3.23E-02 | (Chi2) | 2.22E-16 |
| SEER stage | 2.09E+02 | (Chi2) | 1.70E-16 |
| Invasion level | 1.52E+02 | (Chi2) | 7.29E-17 |
| Ulcer | 7.18E+01 | (Chi2) | 1.73E-16 |
| Reg LN examined | 1.34E+00 | (Chi2) | 8.33E-17 |
| Treatment | 1.25E+02 | (Chi2) | 2.26E-16 |

**Table S2**. Balance check for exact matching of Solitary CMM and CMM with multiple tumors patients. Multivariate Imbalance Measure: L1=0.000; Percentage of local common support: LCS=100%; In the total of 136,823 solitary CMM and 36,693 CMM with multiple tumors, 26,325 were matched.

Abbreviations: Reg, regional; LN, lymph node; SLN, sentinel lymph node
