# Supplementary material for: Characteristics, Prognosis, and Competing Risk Nomograms of Cutaneous Malignant Melanoma: Evidence for Pigmentary Disorders
Source: Front Oncol. 2022 Jun 1;12:838840. doi: 10.3389/fonc.2022.838840 (PMC9198425; doi:10.3389/fonc.2022.838840)
Supplement: Supplementary file 4 [file Table_3.docx]

|  | Subgroups | | | | | | | |  |
| --- | --- | --- | --- | --- | --- | --- | --- | --- | --- |
|  | ℓ1 | ℓ2 | ℓ3 | ℓ4 | ℓ5 | ℓ6 | ℓ7 | ℓ8 |  |
| $\delta$ | D1 | D2 | D3 | D4 | D5 | D6 | D7 | D8 | Dmax |
| -0.0956 | -1.5 | -0.71 | -3.62 | -1.52 | -3.3 | -3.38 | -3.01 | -0.98 | 9.65 |
| -0.0903 | -1.74 | -0.81 | -4.52 | -1.98 | -4.08 | -4.13 | -3.66 | -1.33 | 8.75 |
| -0.0851 | -1.97 | -1 | -5.4 | -2.43 | -4.83 | -4.86 | -4.32 | -1.69 | 7.88 |
| -0.0798 | -2.2 | -1.19 | -6.34 | -2.92 | -5.64 | -5.65 | -5.02 | -2.09 | 7 |
| -0.0746 | -2.43 | -1.28 | -7.22 | -3.38 | -6.39 | -6.39 | -5.67 | -2.45 | 7.72 |
|  | Subgroups | | | | | | | | Total |
| Proportion (%) | 3.9 | 1.5 | 13.8 | 9.2 | 20.7 | 8.3 | 1.8 | 1.8 | 100 |
| Solitary CMM (%) | 2.9 | 2.3 | 0.9 | 1.2 | 1.3 | 1.4 | 4.4 | 2.6 | 1.0 |
| Multiple CMM (%) | 15.8 | 14.4 | 12.2 | 12.0 | 13.7 | 13.2 | 14.2 | 3.2 | 9.5 |
| Risk Difference (%) | -0.1 | -0.1 | -0.1 | -0.1 | -0.1 | -0.1 | -0.1 | 0.0 | -8.5 |
| Odds ratio | 0.2 | 0.1 | 0.1 | 0.1 | 0.1 | 0.1 | 0.3 | 0.8 | 0.1 |

|  | Subgroups | | | | | | |  |
| --- | --- | --- | --- | --- | --- | --- | --- | --- |
|  | ℓ9 | ℓ10 | ℓ11 | ℓ12 | ℓ1_2 | ℓ45 | ℓ10_11 |  |
| $\delta$ | D9 | D10 | D11 | D12 | D13 | D14 | D15 | Dmax |
| -0.0956 | -2.96 | 2.38 | 1.42 | 7.28 | -1.66 | -3.01 | 2.77 | 9.65 |
| -0.0903 | -3.51 | 2.05 | 1.25 | 6.76 | -1.9 | -3.66 | 2.41 | 8.75 |
| -0.0851 | -4.05 | 1.73 | 1 | 6.28 | -2.2 | -4.32 | 2 | 7.88 |
| -0.0798 | -4.63 | 1.41 | 0.84 | 5.77 | -2.5 | -5.02 | 1.63 | 7 |
| -0.0746 | -5.17 | 1.03 | 0.59 | 5.29 | -2.74 | -5.67 | 1.18 | 7.72 |
|  | Subgroups | | | | | | | Total |
| Proportion (%) | 18.9 | 5.8 | 2.5 | 12.1 | 5.4 | 29.8 | 8.3 | 100 |
| Solitary CMM (%) | 0.4 | 0.3 | 0.0 | 0.1 | 2.7 | 1.3 | 0.2 | 1.0 |
| Multiple CMM (%) | 4.9 | 6.1 | 6.0 | 2 | 15.4 | 13.2 | 6.1 | 9.5 |
| Risk Difference (%) | 0.0 | -0.1 | -0.1 | 0 | -0.1 | -0.1 | -0.1 | -8.5 |
| Odds ratio | 0.1 | 0.1 | 0.0 | 0 | 0.2 | 0.1 | 0 | 0.1 |

|  | Subgroups | | | | | | |  |
| --- | --- | --- | --- | --- | --- | --- | --- | --- |
|  | ℓ456 | ℓ9_10_11 | ℓ4567 | ℓ8_9_10_11 | ℓ34567 | ℓ8_9_10_11_12 | ℓ1234567 |  |
| $\delta$ | D16 | D17 | D18 | D19 | D20 | D21 | D22 | Dmax |
| -0.0956 | -3.38 | 6.29 | -3.3 | 6.85 | -3.62 | 9.65 | -3.96 | 9.65 |
| -0.0903 | -4.13 | 5.58 | -4.08 | 6.12 | -4.52 | 8.75 | -4.89 | 8.75 |
| -0.0851 | -4.86 | 4.87 | -4.83 | 5.38 | -5.4 | 7.88 | -5.82 | 7.88 |
| -0.0798 | -5.65 | 4.16 | -5.64 | 4.67 | -6.34 | 7 | -6.81 | 7 |
| -0.0746 | -6.39 | 3.4 | -6.39 | 3.89 | -7.22 | 6.08 | -7.72 | 7.72 |
|  | Subgroups | | | | | | | Total |
| Proportion (%) | 38.1 | 27.1 | 39.9 | 28.9 | 53.7 | 40.9 | 59.1 | 100 |
| Solitary CMM (%) | 1.3 | 0.3 | 1.4 | 0.5 | 1.3 | 0.4 | 1.4 | 1.0 |
| Multiple CMM (%) | 13.2 | 5.3 | 13.2 | 5.1 | 12.9 | 4.2 | 13.2 | 9.5 |
| Risk Difference (%) | -0.1 | 0 | -0.1 | 0.0 | -0.1 | 0 | -0.1 | -8.5 |
| Odds ratio | 0.1 | 0.1 | 0.1 | 0.1 | 0.1 | 0.1 | 0.1 | 0.1 |

**Table S3**. McNemar tests of the null hypothesis of no effect modification for solitary CMM and CMM with multiple tumors on patients dying of other cancers. The upper table shows 22 deviates from the subgroups with the maximum absolute deviate where the critical values $D_{T}=3.03$ when α = 0.04 and γ = 0.01.
