# Supplementary material for: Characteristics, Prognosis, and Competing Risk Nomograms of Cutaneous Malignant Melanoma: Evidence for Pigmentary Disorders
Source: Front Oncol. 2022 Jun 1;12:838840. doi: 10.3389/fonc.2022.838840 (PMC9198425; doi:10.3389/fonc.2022.838840)
Supplement: Supplementary file 5 [file Table_4.docx]

|  | Subgroups | | | | | | | | |  |
| --- | --- | --- | --- | --- | --- | --- | --- | --- | --- | --- |
|  | ℓ1 | ℓ2 | ℓ3 | ℓ4 | ℓ5 | ℓ6 | ℓ7 | ℓ8 | ℓ9 |  |
| $\delta$ | D1 | D2 | D3 | D4 | D5 | D6 | D7 | D8 | D9 | Dmax |
| 0.0163 | -2.87 | 1.86 | 1.09 | 1.9 | 0.67 | 1.78 | 2.86 | 2.95 | 3.03 | 5.05 |
| 0.0218 | -3.84 | 1.42 | 0.81 | 1.46 | 0.51 | 1.38 | 2.57 | 2.43 | 2.63 | 4.13 |
| 0.0274 | -4.78 | 0.99 | 0.53 | 1.03 | 0.35 | 0.97 | 2.33 | 1.93 | 2.22 | 4.78 |
| 0.0329 | -5.73 | 0.55 | 0.21 | 0.6 | 0.2 | 0.57 | 2.04 | 1.38 | 1.82 | 5.73 |
| 0.0384 | -6.68 | 0.11 | -0.07 | 0.2 | 0.04 | 0.2 | 1.8 | 0.86 | 1.41 | 6.68 |
|  | Subgroups | | | | | | | | | Total |
| Proportion (%) | 41.4 | 12.7 | 5.7 | 1.8 | 10.7 | 5 | 11.9 | 2.6 | 5.6 | 100 |
| Solitary CMM (%) | 1.8 | 13.9 | 13.9 | 17.7 | 15.0 | 21.2 | 18.8 | 20.1 | 17.7 | 10.8 |
| Multiple CMM (%) | 1.8 | 9.9 | 10.2 | 13.7 | 10.9 | 13.5 | 13.0 | 13.8 | 15.8 | 7.9 |
| Risk Difference (%) | 0.0 | 0.0 | 0.0 | 0.0 | 0.0 | 0.1 | 0.1 | 0.1 | 0.0 | 2.9 |
| Odds ratio | 1.0 | 1.5 | 1.4 | 1.4 | 1.4 | 1.7 | 1.5 | 1.6 | 1.1 | 1.7 |

|  | Subgroups | | | | | | | | |  |
| --- | --- | --- | --- | --- | --- | --- | --- | --- | --- | --- |
|  | ℓ10 | ℓ23 | ℓ45 | ℓ89 | ℓ789 | ℓ2345 | ℓ23456 | ℓ23456789 | ℓ2345678910 |  |
| $\delta$ | D10 | D11 | D12 | D13 | D14 | D15 | D16 | D17 | D18 | Dmax |
| 0.0163 | 0.98 | 2.15 | 1.86 | 0.98 | 2.95 | 2.87 | 3.74 | 4.77 | 5.05 | 5.05 |
| 0.0218 | 0.65 | 1.64 | 1.42 | 0.65 | 2.43 | 2.19 | 3.01 | 3.86 | 4.13 | 4.13 |
| 0.0274 | 0.35 | 1.12 | 0.99 | 0.35 | 1.93 | 1.52 | 2.29 | 2.99 | 3.24 | 4.78 |
| 0.0329 | -0.02 | 0.57 | 0.55 | -0.02 | 1.38 | 0.82 | 1.54 | 2.06 | 2.29 | 5.73 |
| 0.0384 | -0.35 | 0.05 | 0.11 | -0.35 | 0.86 | 0.17 | 0.84 | 1.19 | 1.41 | 6.68 |
|  | Subgroups | | | | | | | | | Total |
| Proportion (%) | 2.6 | 18.4 | 12.5 | 8.2 | 20.1 | 30.9 | 35.9 | 56 | 58.6 | 100 |
| Solitary CMM (%) | 21.6 | 13.9 | 15.4 | 18.5 | 18.7 | 14.5 | 15.4 | 16.6 | 16.8 | 10.8 |
| Multiple CMM (%) | 14.5 | 10.0 | 11.3 | 15.2 | 13.9 | 10.5 | 10.9 | 12.0 | 12.1 | 7.9 |
| Risk Difference (%) | 0.1 | 0.0 | 0.0 | 0.0 | 0.0 | 0.0 | 0.0 | 0.0 | 0.0 | 2.9 |
| Odds ratio | 1.6 | 1.5 | 1.4 | 1.3 | 1.4 | 1.4 | 1.5 | 1.5 | 1.5 | 1.7 |

**Table S4**. McNemar tests of the null hypothesis of no effect modification for solitary CMM and CMM with multiple tumors on patients dying of noncancerous diseases. The upper table shows 18 deviates from the subgroups with the maximum absolute deviate where the critical values $D_{T}=2.98$ when α = 0.04 and γ = 0.01.
