# Supplementary material for: Characteristics, Prognosis, and Competing Risk Nomograms of Cutaneous Malignant Melanoma: Evidence for Pigmentary Disorders
Source: Front Oncol. 2022 Jun 1;12:838840. doi: 10.3389/fonc.2022.838840 (PMC9198425; doi:10.3389/fonc.2022.838840)
Supplement: Supplementary file 6 [file Table_5.docx]

| Characteristics | Univariate analysis | | Multivariate analysis | |
| --- | --- | --- | --- | --- |
|  | HR (95% CI) | *p*-value | HR (95% CI) | *p*-value |
| **Age** |  |  |  |  |
| Young | Ref |  | Ref |  |
| Middle | 1.48(1.37,1.59) | <0.001 | 1.29(1.19,1.4) | <0.001 |
| Old | 2.3(2.15,2.47) | <0.001 | 1.61(1.5,1.74) | <0.001 |
| **Gender** |  |  |  |  |
| Male | Ref |  | Ref |  |
| Female | 0.56(0.53,0.59) | <0.001 | 0.75(0.71,0.79) | <0.001 |
| **Race** |  |  |  |  |
| White | Ref |  |  |  |
| Black | 3.38(2.8,4.07) | <0.001 |  |  |
| Others | 1.77(1.5,2.09) | <0.001 |  |  |
| **UV exposure** |  |  |  |  |
| High | Ref |  |  |  |
| Low | 0.93(0.88,0.98) | 0.01 |  |  |
| **Ulcer** |  |  |  |  |
| No | Ref |  | Ref |  |
| Yes | 7.7(7.34,8.08) | <0.001 | 1.86(1.75,1.98) | <0.001 |
| **Tumor Thickness** |  |  |  |  |
| ≤100mm | Ref |  |  |  |
| 100-200mm | 6.51(6.04,7.02) | <0.001 |  |  |
| 200-400mm | 12.82(11.84,13.88) | <0.001 |  |  |
| >400mm | 29.8(27.67,32.1) | <0.001 |  |  |
| **AJCC-T Stage** |  |  |  |  |
| T1 | Ref |  | Ref |  |
| T2 | 6.55(6.07,7.07) | <0.001 | 2.37(2.15,2.61) | <0.001 |
| T3 | 12.72(11.74,13.77) | <0.001 | 2.4(2.14,2.7) | <0.001 |
| T4 | 29.8(27.67,32.1) | <0.001 | 3.09(2.74,3.49) | <0.001 |
| **AJCC-N Stage** |  |  |  |  |
| N0 | Ref |  | Ref |  |
| N1 | 7.9(7.41,8.43) | <0.001 | 1.33(1.19,1.5) | <0.001 |
| N2 | 10.31(9.54,11.15) | <0.001 | 1.59(1.41,1.81) | <0.001 |
| N3 | 18.86(17.26,20.62) | <0.001 | 1.95(1.7,2.23) | <0.001 |
| **AJCC-M Stage** |  |  |  |  |
| M0 | Ref |  | Ref |  |
| M1 | 22.87(21.28,24.59) | <0.001 | 2.3(1.92,2.76) | <0.001 |
| **Reg LN examined** |  |  |  |  |
| No | Ref |  |  |  |
| Yes | 2.92(2.78,3.07) | <0.001 |  |  |
| **SLN biopsy** |  |  |  |  |
| No | Ref |  | Ref |  |
| Yes | 1.39(1.33,1.47) | <0.001 | 0.81(0.76,0.85) | <0.001 |
| **Subtype** |  |  |  |  |
| Acral lentiginous | Ref |  | Ref |  |
| Amelanotic | 0.89(0.67,1.2) | 0.45 | 0.88(0.63,1.22) | 0.43 |
| Lentigo | 0.08(0.06,0.1) | <0.001 | 0.46(0.36,0.6) | <0.001 |
| Nodular | 1.16(1,1.34) | 0.04 | 0.84(0.71,0.99) | 0.04 |
| Superficial spreading | 0.14(0.12,0.16) | <0.001 | 0.56(0.48,0.67) | <0.001 |
| Other uncommon types | 0.34(0.3,0.39) | <0.001 | 0.75(0.64,0.88) | <0.001 |
| **Invasion level** |  |  |  |  |
| Ⅱ | Ref |  | Ref |  |
| Ⅲ | 3.79(3.3,4.35) | <0.001 | 3.07(2.67,3.53) | <0.001 |
| Ⅳ | 16.92(14.99,19.09) | <0.001 | 4.99(4.3,5.78) | <0.001 |
| Ⅴ | 73.89(65.19,83.75) | <0.001 | 4.78(4.01,5.7) | <0.001 |
| **SEER stage** |  |  |  |  |
| Localized | Ref |  | Ref |  |
| Regional | 10.23(9.69,10.8) | <0.001 | 2.07(1.82,2.35) | <0.001 |
| Distant | 32.55(30.35,34.91) | <0.001 | 2.46(2.02,3.01) | <0.001 |
| **Treatment** |  |  |  |  |
| No treatment | Ref |  | Ref |  |
| Surgery only | 0.31(0.28,0.34) | <0.001 | 0.6(0.53,0.68) | <0.001 |
| CT | 4.49(3.99,5.06) | <0.001 | 1.19(1.03,1.39) | 0.021 |
| RT | 3.62(3.19,4.11) | <0.001 | 1.05(0.9,1.23) | 0.54 |
| CT and RT | 7.87(6.59,9.38) | <0.001 | 1.49(1.19,1.86) | <0.001 |
| **Laterality** |  |  |  |  |
| one side | Ref |  |  |  |
| paired sides | 0.95(0.87,1.03) | 0.23 |  |  |

**Table S5**. Univariate and multivariate analyses by Fine–Gray proportional sub-distribution hazards model for patient death of CMM among patients with solitary CMM. Age: young (≤45 years), middle (45-60 years), old (>60 years).

Abbreviations: Reg, regional; LN, lymph node; SLN, sentinel lymph node; CT, chemotherapy (with/without surgery); RT, radiotherapy (with/without surgery); CT and RT, chemotherapy and radiotherapy (with/without surgery); CI, confidence interval; HR, hazard ratio; Ref, reference.
