# Supplementary material for: Characteristics, Prognosis, and Competing Risk Nomograms of Cutaneous Malignant Melanoma: Evidence for Pigmentary Disorders
Source: Front Oncol. 2022 Jun 1;12:838840. doi: 10.3389/fonc.2022.838840 (PMC9198425; doi:10.3389/fonc.2022.838840)
Supplement: Supplementary file 7 [file Table_6.docx]

| Characteristics | Univariate analysis | | Multivariate analysis | |
| --- | --- | --- | --- | --- |
|  | HR (95% CI) | *p*-value | HR (95% CI) | *p*-value |
| **Age** |  |  |  |  |
| Young | Ref |  |  |  |
| Middle | 1.29(1.01,1.63) | 0.038 |  |  |
| Old | 1.65(1.33,2.06) | <0.001 |  |  |
| **Gender** |  |  |  |  |
| Male | Ref |  | Ref |  |
| Female | 0.67(0.59,0.75) | <0.001 | 0.79(0.69,0.9) | <0.001 |
| **Race** |  |  |  |  |
| White | Ref |  |  |  |
| Black | 2.47(1.32,4.61) | 0.005 |  |  |
| Others | 1.81(1.03,3.2) | 0.041 |  |  |
| **UV exposure** |  |  |  |  |
| High | Ref |  |  |  |
| Low | 0.95(0.84,1.07) | 0.41 |  |  |
| **Ulcer** |  |  |  |  |
| No | Ref |  | Ref |  |
| Yes | 5.39(4.8,6.04) | <0.001 | 1.85(1.6,2.13) | <0.001 |
| **Tumor Thickness** |  |  |  |  |
| ≤100mm | Ref |  | Ref |  |
| 100-200mm | 3.64(3.12,4.26) | <0.001 | 1.91(1.58,2.31) | <0.001 |
| 200-400mm | 7.92(6.74,9.3) | <0.001 | 2.66(2.14,3.3) | <0.001 |
| >400mm | 12.86(10.9,15.16) | <0.001 | 3.08(2.39,3.97) | <0.001 |
| **AJCC-T Stage** |  |  |  |  |
| T1 | Ref |  |  |  |
| T2 | 3.64(3.12,4.26) | <0.001 |  |  |
| T3 | 7.93(6.75,9.31) | <0.001 |  |  |
| T4 | 12.86(10.9,15.16) | <0.001 |  |  |
| **AJCC-N Stage** |  |  |  |  |
| N0 | Ref |  | Ref |  |
| N1 | 5.48(4.64,6.49) | <0.001 | 2.03(1.67,2.46) | <0.001 |
| N2 | 6.79(5.42,8.5) | <0.001 | 2.23(1.75,2.84) | <0.001 |
| N3 | 14.27(11.09,18.37) | <0.001 | 3.77(2.81,5.05) | <0.001 |
| **AJCC-M Stage** |  |  |  |  |
| M0 | Ref |  | Ref |  |
| M1 | 10.56(7.99,13.96) | <0.001 | 2.07(1.43,2.99) | <0.001 |
| **Reg LN examined** |  |  |  |  |
| No | Ref |  |  |  |
| Yes | 3.01(2.68,3.38) | <0.001 |  |  |
| **SLN biopsy** |  |  |  |  |
| No | Ref |  |  |  |
| Yes | 1.97(1.75,2.2) | <0.001 |  |  |
| **Subtype** |  |  |  |  |
| Acral lentiginous | Ref |  |  |  |
| Amelanotic | 0.78(0.35,1.74) | 0.55 |  |  |
| Lentigo | 0.22(0.13,0.36) | <0.001 |  |  |
| Nodular | 1.47(0.98,2.22) | 0.066 |  |  |
| Superficial spreading | 0.32(0.21,0.48) | <0.001 |  |  |
| Other uncommon types | 0.43(0.29,0.64) | <0.001 |  |  |
| **Invasion level** |  |  |  |  |
| Ⅱ | Ref |  | Ref |  |
| Ⅲ | 2.57(2.04,3.24) | <0.001 | 2.04(1.61,2.59) | <0.001 |
| Ⅳ | 7.88(6.48,9.59) | <0.001 | 2.96(2.32,3.77) | <0.001 |
| Ⅴ | 19.07(15.09,24.09) | <0.001 | 3.39(2.46,4.67) | <0.001 |
| **SEER stage** |  |  |  |  |
| Localized | Ref |  |  |  |
| Regional | 6.04(5.32,6.86) | <0.001 |  |  |
| Distant | 11.39(8.94,14.52) | <0.001 |  |  |
| **Treatment** |  |  |  |  |
| No treatment | Ref |  | Ref |  |
| Surgery only | 0.58(0.44,0.76) | <0.001 | 0.54(0.4,0.74) | <0.001 |
| CT | 3.56(2.36,5.35) | <0.001 | 0.78(0.5,1.23) | 0.29 |
| RT | 3.77(2.55,5.58) | <0.001 | 0.8(0.5,1.26) | 0.33 |
| CT and RT | 17.91(9.63,33.29) | <0.001 | 3.3(1.28,8.53) | 0.014 |
| **Laterality** |  |  |  |  |
| one side | Ref |  |  |  |
| paired sides | 0.95(0.77,1.18) | 0.67 |  |  |

**Table S6**. Univariate and multivariate analyses by Fine–Gray proportional sub-distribution hazards model for patient death of CMM among patients with CMM with multiple tumors. Age: young (≤45 years), middle (45-60 years), old (>60 years).

Abbreviations: Reg, regional; LN, lymph node; SLN, sentinel lymph node; CT, chemotherapy (with/without surgery); RT, radiotherapy (with/without surgery); CT and RT, chemotherapy and radiotherapy (with/without surgery); CI, confidence interval; HR, hazard ratio; Ref, reference.
