# Supplementary material for: Characteristics, Prognosis, and Competing Risk Nomograms of Cutaneous Malignant Melanoma: Evidence for Pigmentary Disorders
Source: Front Oncol. 2022 Jun 1;12:838840. doi: 10.3389/fonc.2022.838840 (PMC9198425; doi:10.3389/fonc.2022.838840)
Supplement: Supplementary file 8 [file Table_7.docx]

| Characteristics | Univariate analysis | | Multivariate analysis | |
| --- | --- | --- | --- | --- |
|  | HR (95% CI) | *p*-value | HR (95% CI) | *p*-value |
| **Age** |  |  |  |  |
| Young | Ref |  | Ref |  |
| Middle | 1.86(1.41,2.45) | <0.001 | 1.62(1.23,2.14) | <0.001 |
| Old | 5.47(4.29,6.98) | <0.001 | 3.64(2.83,4.68) | <0.001 |
| **Gender** |  |  |  |  |
| Male | Ref |  |  |  |
| Female | 0.6(0.53,0.69) | <0.001 |  |  |
| **Race** |  |  |  |  |
| White | Ref |  |  |  |
| Black | 3.35(2.07,5.42) | <0.001 |  |  |
| Others | 1.03(0.58,1.82) | 0.92 |  |  |
| **UV exposure** |  |  |  |  |
| High | Ref |  | Ref |  |
| Low | 0.75(0.65,0.87) | <0.001 | 0.77(0.66,0.9) | <0.001 |
| **Ulcer** |  |  |  |  |
| No | Ref |  | Ref |  |
| Yes | 5.34(4.69,6.08) | <0.001 | 1.93(1.64,2.28) | <0.001 |
| **Tumor Thickness** |  |  |  |  |
| ≤100mm | Ref |  | Ref |  |
| 100-200mm | 3.54(2.96,4.22) | <0.001 | 1.93(1.54,2.42) | <0.001 |
| 200-400mm | 5.33(4.35,6.53) | <0.001 | 1.99(1.51,2.63) | <0.001 |
| >400mm | 12.26(10.31,14.59) | <0.001 | 2.44(1.84,3.24) | <0.001 |
| **AJCC-T Stage** |  |  |  |  |
| T1 | Ref |  |  |  |
| T2 | 3.56(2.98,4.25) | <0.001 |  |  |
| T3 | 5.27(4.3,6.47) | <0.001 |  |  |
| T4 | 12.26(10.31,14.59) | <0.001 |  |  |
| **AJCC-N Stage** |  |  |  |  |
| N0 | Ref |  |  |  |
| N1 | 3.04(2.46,3.74) | <0.001 |  |  |
| N2 | 3.31(2.5,4.39) | <0.001 |  |  |
| N3 | 4.52(3.31,6.18) | <0.001 |  |  |
| **AJCC-M Stage** |  |  |  |  |
| M0 | Ref |  | Ref |  |
| M1 | 10.95(9.24,12.98) | <0.001 | 1.87(1.47,2.38) | <0.001 |
| **Reg LN examined** |  |  |  |  |
| No | Ref |  |  |  |
| Yes | 1.21(1.06,1.38) | 0.005 |  |  |
| **SLN biopsy** |  |  |  |  |
| No | Ref |  | Ref |  |
| Yes | 0.8(0.68,0.93) | 0.005 | 0.57(0.48,0.67) | <0.001 |
| **Subtype** |  |  |  |  |
| Acral lentiginous | Ref |  |  |  |
| Amelanotic | 0.9(0.33,2.42) | 0.83 |  |  |
| Lentigo | 0.33(0.19,0.6) | <0.001 |  |  |
| Nodular | 1.3(0.79,2.15) | 0.31 |  |  |
| Superficial spreading | 0.25(0.15,0.42) | <0.001 |  |  |
| Other uncommon types | 0.64(0.4,1.04) | 0.07 |  |  |
| **Invasion level** |  |  |  |  |
| Ⅱ | Ref |  | Ref |  |
| Ⅲ | 1.83(1.41,2.39) | <0.001 | 1.78(1.36,2.31) | <0.001 |
| Ⅳ | 5.3(4.28,6.58) | <0.001 | 2.77(2.11,3.62) | <0.001 |
| Ⅴ | 19.14(15.21,24.08) | <0.001 | 3.96(2.86,5.48) | <0.001 |
| **SEER stage** |  |  |  |  |
| Localized | Ref |  |  |  |
| Regional | 4.04(3.43,4.75) | <0.001 |  |  |
| Distant | 12.26(10.35,14.51) | <0.001 |  |  |
| **Treatment** |  |  |  |  |
| No treatment | Ref |  | Ref |  |
| Surgery only | 0.2(0.17,0.24) | <0.001 | 0.45(0.37,0.56) | <0.001 |
| CT | 1.18(0.86,1.62) | 0.3 | 0.7(0.5,0.97) | 0.031 |
| RT | 1.16(0.83,1.62) | 0.4 | 0.66(0.46,0.93) | 0.018 |
| CT and RT | 1.58(0.97,2.57) | 0.064 | 0.86(0.52,1.42) | 0.55 |
| **Laterality** |  |  |  |  |
| one side | Ref |  |  |  |
| paired sides | 1.16(0.93,1.44) | 0.19 |  |  |

**Table S7**. Univariate and multivariate analyses by Fine–Gray proportional sub-distribution hazards model for patient death of other cancers among patients with solitary CMM. Age: young (≤45 years), middle (45-60 years), old (>60 years).

Abbreviations: Reg, regional; LN, lymph node; SLN, sentinel lymph node; CT, chemotherapy (with/without surgery); RT, radiotherapy (with/without surgery); CT and RT, chemotherapy and radiotherapy (with/without surgery); CI, confidence interval; HR, hazard ratio; Ref, reference.
