# Supplementary material for: Characteristics, Prognosis, and Competing Risk Nomograms of Cutaneous Malignant Melanoma: Evidence for Pigmentary Disorders
Source: Front Oncol. 2022 Jun 1;12:838840. doi: 10.3389/fonc.2022.838840 (PMC9198425; doi:10.3389/fonc.2022.838840)
Supplement: Supplementary file 9 [file Table_8.docx]

| Characteristics | Univariate analysis | | Multivariate analysis | |
| --- | --- | --- | --- | --- |
|  | HR (95% CI) | *p*-value | HR (95% CI) | *p*-value |
| **Age** |  |  |  |  |
| Young | Ref |  | Ref |  |
| Middle | 2.45(1.84,3.25) | <0.001 | 2.38(1.79,3.17) | <0.001 |
| Old | 6.5(4.97,8.5) | <0.001 | 6.04(4.61,7.9) | <0.001 |
| **Gender** |  |  |  |  |
| Male | Ref |  |  |  |
| Female | 0.83(0.75,0.9) | <0.001 |  |  |
| **Race** |  |  |  |  |
| White | Ref |  |  |  |
| Black | 1.14(0.59,2.21) | 0.69 |  |  |
| Others | 1.04(0.6,1.8) | 0.9 |  |  |
| **UV exposure** |  |  |  |  |
| High | Ref |  |  |  |
| Low | 0.9(0.82,0.99) | 0.024 |  |  |
| **Ulcer** |  |  |  |  |
| No | Ref |  |  |  |
| Yes | 1.43(1.28,1.6) | <0.001 |  |  |
| **Tumor Thickness** |  |  |  |  |
| ≤100mm | Ref |  | Ref |  |
| 100-200mm | 1.27(1.15,1.41) | <0.001 | 1.2(1.07,1.35) | 0.003 |
| 200-400mm | 1.5(1.31,1.72) | <0.001 | 1.34(1.13,1.58) | <0.001 |
| >400mm | 1.82(1.56,2.13) | <0.001 | 1.42(1.16,1.73) | <0.001 |
| **AJCC-T Stage** |  |  |  |  |
| T1 | Ref |  |  |  |
| T2 | 1.27(1.15,1.41) | <0.001 |  |  |
| T3 | 1.5(1.31,1.72) | <0.001 |  |  |
| T4 | 1.82(1.56,2.13) | <0.001 |  |  |
| **AJCC-N Stage** |  |  |  |  |
| N0 | Ref |  |  |  |
| N1 | 0.94(0.75,1.18) | 0.58 |  |  |
| N2 | 1.16(0.85,1.58) | 0.34 |  |  |
| N3 | 0.73(0.42,1.27) | 0.27 |  |  |
| **AJCC-M Stage** |  |  |  |  |
| M0 | Ref |  |  |  |
| M1 | 1.58(1.08,2.33) | 0.02 |  |  |
| **Reg LN examined** |  |  |  |  |
| No | Ref |  | Ref |  |
| Yes | 0.91(0.83,1) | 0.042 | 0.71(0.64,0.79) | <0.001 |
| **SLN biopsy** |  |  |  |  |
| No | Ref |  |  |  |
| Yes | 0.92(0.84,1.02) | 0.11 |  |  |
| **Subtype** |  |  |  |  |
| Acral lentiginous | Ref |  |  |  |
| Amelanotic | 1.01(0.52,1.97) | 0.97 |  |  |
| Lentigo | 0.82(0.56,1.22) | 0.33 |  |  |
| Nodular | 0.88(0.6,1.31) | 0.54 |  |  |
| Superficial spreading | 0.55(0.38,0.8) | 0.002 |  |  |
| Other uncommon types | 0.66(0.46,0.96) | 0.029 |  |  |
| **Invasion level** |  |  |  |  |
| Ⅱ | Ref |  | Ref |  |
| Ⅲ | 1.05(0.94,1.18) | 0.37 | 1.14(1.02,1.28) | 0.025 |
| Ⅳ | 1.38(1.25,1.52) | <0.001 | 1.38(1.21,1.57) | <0.001 |
| Ⅴ | 2.09(1.74,2.5) | <0.001 | 1.71(1.36,2.15) | <0.001 |
| **SEER stage** |  |  |  |  |
| Localized | Ref |  |  |  |
| Regional | 1.27(1.1,1.47) | 0.001 |  |  |
| Distant | 1.64(1.19,2.25) | 0.002 |  |  |
| **Treatment** |  |  |  |  |
| No treatment | Ref |  |  |  |
| Surgery only | 0.72(0.58,0.9) | 0.004 |  |  |
| CT | 0.39(0.2,0.76) | 0.006 |  |  |
| RT | 0.84(0.52,1.36) | 0.49 |  |  |
| CT and RT | 0.45(0.11,1.89) | 0.27 |  |  |
| **Laterality** |  |  |  |  |
| one side | Ref |  |  |  |
| paired sides | 1.07(0.92,1.24) | 0.37 |  |  |

**Table S8**. Univariate and multivariate analyses by Fine–Gray proportional sub-distribution hazards model for patient death of other cancers among patients with CMM with multiple tumors. Age: young (≤45 years), middle (45-60 years), old (>60 years).

Abbreviations: Reg, regional; LN, lymph node; SLN, sentinel lymph node; CT, chemotherapy (with/without surgery); RT, radiotherapy (with/without surgery); CT and RT, chemotherapy and radiotherapy (with/without surgery); CI, confidence interval; HR, hazard ratio; Ref, reference.
