# Supplementary material for: Characteristics, Prognosis, and Competing Risk Nomograms of Cutaneous Malignant Melanoma: Evidence for Pigmentary Disorders
Source: Front Oncol. 2022 Jun 1;12:838840. doi: 10.3389/fonc.2022.838840 (PMC9198425; doi:10.3389/fonc.2022.838840)
Supplement: Supplementary file 10 [file Table_9.docx]

| Characteristics | Univariate analysis | | Multivariate analysis | |
| --- | --- | --- | --- | --- |
|  | HR (95% CI) | *p*-value | HR (95% CI) | *p*-value |
| **Age** |  |  |  |  |
| Young | Ref |  | Ref |  |
| Middle | 3.06(2.59,3.63) | <0.001 | 2.85(2.41,3.37) | <0.001 |
| Old | 28.56(24.53,33.24) | <0.001 | 22.6(19.38,26.34) | <0.001 |
| **Gender** |  |  |  |  |
| Male | Ref |  | Ref |  |
| Female | 0.64(0.61,0.67) | <0.001 | 0.8(0.76,0.84) | <0.001 |
| **Race** |  |  |  |  |
| White | Ref |  |  |  |
| Black | 1.1(0.83,1.46) | 0.51 |  |  |
| Others | 0.44(0.33,0.6) | <0.001 |  |  |
| **UV exposure** |  |  |  |  |
| High | Ref |  |  |  |
| Low | 0.92(0.87,0.96) | <0.001 |  |  |
| **Ulcer** |  |  |  |  |
| No | Ref |  | Ref |  |
| Yes | 1.94(1.84,2.05) | <0.001 | 1.29(1.2,1.38) | <0.001 |
| **Tumor Thickness** |  |  |  |  |
| ≤100mm | Ref |  |  |  |
| 100-200mm | 1.32(1.25,1.39) | <0.001 |  |  |
| 200-400mm | 2.02(1.88,2.16) | <0.001 |  |  |
| >400mm | 2.22(2.06,2.39) | <0.001 |  |  |
| **AJCC-T Stage** |  |  |  |  |
| T1 | Ref |  | Ref |  |
| T2 | 1.32(1.25,1.39) | <0.001 | 1.22(1.14,1.31) | <0.001 |
| T3 | 2.02(1.89,2.17) | <0.001 | 1.56(1.42,1.71) | <0.001 |
| T4 | 2.22(2.06,2.39) | <0.001 | 1.4(1.26,1.57) | <0.001 |
| **AJCC-N Stage** |  |  |  |  |
| N0 | Ref |  |  |  |
| N1 | 0.73(0.64,0.82) | <0.001 |  |  |
| N2 | 0.82(0.69,0.97) | 0.023 |  |  |
| N3 | 0.86(0.68,1.07) | 0.17 |  |  |
| **AJCC-M Stage** |  |  |  |  |
| M0 | Ref |  | Ref |  |
| M1 | 0.77(0.64,0.92) | 0.004 | 0.36(0.29,0.45) | <0.001 |
| **Reg LN examined** |  |  |  |  |
| No | Ref |  | Ref |  |
| Yes | 0.6(0.57,0.64) | 0.042 | 0.42(0.4,0.45) | <0.001 |
| **SLN biopsy** |  |  |  |  |
| No | Ref |  |  |  |
| Yes | 0.62(0.58,0.65) | <0.001 |  |  |
| **Subtype** |  |  |  |  |
| Acral lentiginous | Ref |  | Ref |  |
| Amelanotic | 1.44(0.97,2.12) | 0.068 | 1.39(0.94,2.05) | 0.1 |
| Lentigo | 2.02(1.61,2.53) | <0.001 | 1.71(1.36,2.16) | <0.001 |
| Nodular | 1.65(1.31,2.07) | <0.001 | 1.44(1.14,1.82) | 0.002 |
| Superficial spreading | 0.67(0.53,0.83) | <0.001 | 1.12(0.89,1.4) | 0.34 |
| Other uncommon types | 0.91(0.73,1.14) | 0.42 | 1.21(0.96,1.51) | 0.1 |
| **Invasion level** |  |  |  |  |
| Ⅱ | Ref |  | Ref |  |
| Ⅲ | 1(0.94,1.07) | 0.88 | 1.18(1.11,1.26) | <0.001 |
| Ⅳ | 1.6(1.51,1.68) | <0.001 | 1.67(1.56,1.79) | <0.001 |
| Ⅴ | 1.93(1.77,2.11) | <0.001 | 1.72(1.52,1.94) | <0.001 |
| **SEER stage** |  |  |  |  |
| Localized | Ref |  |  |  |
| Regional | 1.23(1.14,1.32) | <0.001 |  |  |
| Distant | 0.88(0.75,1.02) | 0.089 |  |  |
| **Treatment** |  |  |  |  |
| No treatment | Ref |  | Ref |  |
| Surgery only | 0.66(0.6,0.73) | <0.001 | 0.89(0.8,0.99) | 0.036 |
| CT | 0.3(0.22,0.41) | <0.001 | 0.43(0.31,0.58) | <0.001 |
| RT | 0.65(0.51,0.82) | <0.001 | 0.58(0.45,0.74) | <0.001 |
| CT and RT | 0.41(0.25,0.67) | <0.001 | 0.63(0.38,1.04) | 0.071 |
| **Laterality** |  |  |  |  |
| one side | Ref |  |  |  |
| paired sides | 1.04(0.96,1.13) | 0.29 |  |  |

**Table S9**. Univariate and multivariate analyses by Fine–Gray proportional sub-distribution hazards model for patient death of noncancers-diseases among patients with solitary CMM. Age: young (≤45 years), middle (45-60 years), old (>60 years).

Abbreviations: Reg, regional; LN, lymph node; SLN, sentinel lymph node; CT, chemotherapy (with/without surgery); RT, radiotherapy (with/without surgery); CT and RT, chemotherapy and radiotherapy (with/without surgery); CI, confidence interval; HR, hazard ratio; Ref, reference.
