# Supplementary material for: Characteristics, Prognosis, and Competing Risk Nomograms of Cutaneous Malignant Melanoma: Evidence for Pigmentary Disorders
Source: Front Oncol. 2022 Jun 1;12:838840. doi: 10.3389/fonc.2022.838840 (PMC9198425; doi:10.3389/fonc.2022.838840)
Supplement: Supplementary file 11 [file Table_10.docx]

| Characteristics | Univariate analysis | | Multivariate analysis | |
| --- | --- | --- | --- | --- |
|  | HR (95% CI) | *p*-value | HR (95% CI) | *p*-value |
| **Age** |  |  |  |  |
| Young | Ref |  | Ref |  |
| Middle | 1.42(0.93,2.17) | 0.1 | 1.34(0.87,2.04) | 0.18 |
| Old | 9.69(6.63,14.18) | <0.001 | 8.49(5.79,12.47) | <0.001 |
| **Gender** |  |  |  |  |
| Male | Ref |  | Ref |  |
| Female | 0.66(0.59,0.74) | <0.001 | 0.82(0.73,0.92) | <0.001 |
| **Race** |  |  |  |  |
| White | Ref |  |  |  |
| Black | 0.94(0.39,2.28) | 0.89 |  |  |
| Others | 0.95(0.47,1.9) | 0.88 |  |  |
| **UV exposure** |  |  |  |  |
| High | Ref |  |  |  |
| Low | 0.9(0.8,1) | 0.055 |  |  |
| **Ulcer** |  |  |  |  |
| No | Ref |  |  |  |
| Yes | 1.48(1.3,1.7) | <0.001 |  |  |
| **Tumor Thickness** |  |  |  |  |
| ≤100mm | Ref |  |  |  |
| 100-200mm | 1.38(1.22,1.56) | <0.001 | 1.56(1.37,1.78) | <0.001 |
| 200-400mm | 1.56(1.32,1.84) | <0.001 | 1.82(1.52,2.18) | <0.001 |
| >400mm | 1.76(1.45,2.13) | <0.001 | 1.9(1.54,2.33) | <0.001 |
| **AJCC-T Stage** |  |  |  |  |
| T1 | Ref |  |  |  |
| T2 | 1.38(1.22,1.56) | <0.001 |  |  |
| T3 | 1.56(1.32,1.84) | <0.001 |  |  |
| T4 | 1.76(1.45,2.13) | <0.001 |  |  |
| **AJCC-N Stage** |  |  |  |  |
| N0 | Ref |  |  |  |
| N1 | 0.51(0.35,0.74) | <0.001 |  |  |
| N2 | 0.97(0.64,1.46) | 0.88 |  |  |
| N3 | 0.85(0.45,1.6) | 0.61 |  |  |
| **AJCC-M Stage** |  |  |  |  |
| M0 | Ref |  |  |  |
| M1 | 0.86(0.47,1.57) | 0.63 |  |  |
| **Reg LN examined** |  |  |  |  |
| No | Ref |  | Ref |  |
| Yes | 0.76(0.68,0.85) | <0.001 | 0.61(0.54,0.7) | <0.001 |
| **SLN biopsy** |  |  |  |  |
| No | Ref |  |  |  |
| Yes | 0.77(0.68,0.87) | <0.001 |  |  |
| **Subtype** |  |  |  |  |
| Acral lentiginous | Ref |  |  |  |
| Amelanotic | 1.03(0.42,2.49) | 0.95 |  |  |
| Lentigo | 1.38(0.82,2.3) | 0.23 |  |  |
| Nodular | 1.15(0.68,1.95) | 0.59 |  |  |
| Superficial spreading | 0.71(0.43,1.17) | 0.18 |  |  |
| Other uncommon types | 0.85(0.52,1.4) | 0.52 |  |  |
| **Invasion level** |  |  |  |  |
| Ⅱ | Ref |  |  |  |
| Ⅲ | 0.98(0.85,1.13) | 0.79 |  |  |
| Ⅳ | 1.38(1.22,1.55) | <0.001 |  |  |
| Ⅴ | 1.78(1.42,2.24) | <0.001 |  |  |
| **SEER stage** |  |  |  |  |
| Localized | Ref |  |  |  |
| Regional | 1.06(0.88,1.28) | 0.52 |  |  |
| Distant | 0.85(0.52,1.4) | 0.52 |  |  |
| **Treatment** |  |  |  |  |
| No treatment | Ref |  |  |  |
| Surgery only | 0.77(0.59,1.01) | 0.062 |  |  |
| CT | 0.5(0.24,1.06) | 0.072 |  |  |
| RT | 0.77(0.41,1.44) | 0.41 |  |  |
| CT and RT | 0(0,0) | <0.001 |  |  |
| **Laterality** |  |  |  |  |
| one side | Ref |  |  |  |
| paired sides | 0.86(0.7,1.05) | 0.13 |  |  |

**Table S10**. Univariate and multivariate analyses by Fine–Gray proportional sub-distribution hazards model for patient death of noncancers-diseases among patients with CMM with multiple tumors. Age: young (≤45 years), middle (45-60 years), old (>60 years).

Abbreviations: Reg, regional; LN, lymph node; SLN, sentinel lymph node; CT, chemotherapy (with/without surgery); RT, radiotherapy (with/without surgery); CT and RT, chemotherapy and radiotherapy (with/without surgery); CI, confidence interval; HR, hazard ratio; Ref, reference.
